# Identification of genomic regions associated with fatty acid metabolism across blood, liver, backfat and muscle in pigs

**DOI:** 10.1186/s12711-024-00933-3

**Published:** 2024-09-26

**Authors:** Junhui Liu, Cristina Sebastià, Teodor Jové-Juncà, Raquel Quintanilla, Olga González-Rodríguez, Magí Passols, Anna Castelló, Armand Sánchez, Maria Ballester, Josep M. Folch

**Affiliations:** 1 Plant and Animal Genomics, Centre for Research in Agricultural Genomics (CRAG), CSIC-IRTA-UAB-UB Consortium, 08193 Bellaterra, Spain; 2 Departament de Ciència Animal i dels Aliments, Facultat de Veterinària, Universitat Autònoma de Barcelona (UAB), 08193 Bellaterra, Spain; 3 Animal Breeding and Genetics Program, Institut de Recerca i Tecnologia Agroalimentàries (IRTA), Torre Marimon, 08140 Caldes de Montbui, Spain

## Abstract

**Background:**

The composition and distribution of fatty acids (FA) are important factors determining the quality, flavor, and nutrient value of meat. In addition, FAs synthesized in the body participate in energy metabolism and are involved in different regulatory pathways in the form of signaling molecules or by acting as agonist or antagonist ligands of different nuclear receptors. Finally, synthesis and catabolism of FAs affect adaptive immunity by regulating lymphocyte metabolism. The present study performed genome-wide association studies using FA profiles of blood, liver, backfat and muscle from 432 commercial Duroc pigs.

**Results:**

Twenty-five genomic regions located on 15 *Sus scrofa* chromosomes (SSC) were detected. Annotation of the quantitative trait locus (QTL) regions identified 49 lipid metabolism-related candidate genes. Among these QTLs, four were identified in more than one tissue. The ratio of C20:4*n*-6/C20:3*n*-6 was associated with the region on SSC2 at 7.56–14.26 Mb for backfat, liver, and muscle. Members of the fatty acid desaturase gene cluster (*FADS1*, *FADS2*, and *FADS3*) are the most promising candidate genes in this region. Two QTL regions on SSC14 (103.81–115.64 Mb and 100.91–128.14 Mb) were identified for FA desaturation in backfat and muscle. In addition, two separate regions on SSC9 at 0 – 14.55 Mb and on SSC12 at 0–1.91 Mb were both associated with the same multiple FA traits for backfat, with candidate genes involved in de novo FA synthesis and triacylglycerol (TAG) metabolism, such as *DGAT2* and *FASN*. The ratio C20:0/C18:0 was associated with the region on SSC5 at 64.84–78.32 Mb for backfat. Furthermore, the association of the C16:0 content with the region at 118.92–123.95 Mb on SSC4 was blood specific. Finally, candidate genes involved in de novo lipogenesis regulate T cell differentiation and promote the generation of palmitoleate, an adipokine that alleviates inflammation.

**Conclusions:**

Several SNPs and candidate genes were associated with lipid metabolism in blood, liver, backfat, and muscle. These results contribute to elucidating the molecular mechanisms implicated in the determination of the FA profile in different pig tissues and can be useful in selection programs that aim to improve health and energy metabolism in pigs.

**Supplementary Information:**

The online version contains supplementary material available at 10.1186/s12711-024-00933-3.

## Background

Genetic selection has greatly improved the productive performance of commercial pigs in the past decades, while immunocompetence and seen less improvement. With the emergence of antibiotic resistance and the increasing demand for healthier livestock products and for more sustainable production systems [1], it is necessary to establish breeding programs that not only prioritize productive traits but also considers health-related traits.

Energy homeostasis plays an important role in organism health and survival. Fatty acids (FAs) are critical substrates for oxidation and for production of cellular energy. They also serve as signaling molecules and precursors of lipid mediators, playing roles in the regulation of immune responses, including the initiation and resolution of inflammation, as well as in leukocyte trafficking and clearance [2]. Therefore, as a part of being important indicators of meat quality and nutritional value [3], they also modulate immune processes and metabolic homeostasis [4].

As early as 1960s, Newsholme reported that FAs suppress the insulin-induced glucose uptake in isolated rat heart muscle, which revealed the connection between peripheral lipids and cell insulin sensitivity [5]. A study in dairy cattle identified that plasma FAs in different lipid classes can serve as biomarkers for early diagnosis of metabolic diseases [6]. In addition, several studies observed that the distribution and composition of FAs strongly affect cell signaling and function. For instance, an elevated level of 20:4*n*-6 (arachidonic acid, AA) in the cell membrane was associated with increased human chronic inflammation, while omega-3 (N3) polyunsaturated FAs (PUFAs) can inhibit T cell proliferation and Th17 cell differentiation [7–9]. T cells are the most important cells in the adaptive immune system and are activated and exert specific defenses by antigen-presenting cells processed antigens via receptors against a variety of intrinsic or extrinsic aggressors [10]. These receptors are embedded in the cell membrane composed of various lipids. Therefore, lipid components may have an impact on the physical, chemical, and functional properties of T-cell receptors, resulting in the well-defined T-cell functions [10]. Overall, these studies suggest that FAs can be used as potential biomarkers to reflect the metabolic or health state of the organism.

A genome-wide association study (GWAS) is a method to explore the genetic determinism for complex traits by the identification of significant quantitative trait loci (QTLs) across multiple populations and important metabolic tissues, and the genomic regions and molecular markers that are useful for genetic selection. The Duroc breed of pigs, which has an excellent growth rate and meat quality and high content of intramuscular fat, is typically used as the terminal sire breed when fattening pigs are produced. Adipose tissue, muscle, and liver are important tissues involved in FA metabolism [11] and can store FAs in the form of TAG or hydrolyze it into FAs. FAs released from adipose tissue can be delivered to other organs by the circulatory system. In muscle, the released FAs are used as the substrate for oxidation, while in liver, FAs can be re-esterified to TAG and secreted as very-low-density lipoprotein [11]. FAs cooperate among tissues and are affected by the energy requirements of the organism, regulated through the pathways described above. For instance, when the organism is in a state of undernutrition or fasting, fat is the preliminary fuel, through an increase in circulating non-esterified FAs (NEFA) from adipose tissue [12]. Moreover, through gluconeogenesis and ketogenesis pathways, the liver facilitates the release of glucose and ketone bodies. Greater levels of NEFA in blood promote the uptake of FAs by the liver, which are then converted to acyl-CoA for either storage as triacylglycerol in lipid droplets or for energy production through oxidation in the mitochondria [13]. When the intake of hepatic FAs exceeds their consumption, acyl-carnitines accumulate and may spill over into the bloodstream, accompanied by the accumulation of acetyl-CoA in mitochondria, which is converted to ketone bodies [13].

The present study aimed to identify the genomic regions associated with FA profiles and metabolic ratios of FAs in blood, liver, adipose tissue, and muscle in Duroc pigs by GWAS, as well as to pinpoint the most relevant polymorphisms, candidate genes, and biological processes involved in energy metabolism. A comprehensive study integrating FA composition data from the main metabolic tissues in pigs expands our knowledge of genes and metabolic pathways involved in energy homeostasis, providing insights into how energy metabolism affects production-quality and immunity traits in pigs. This will help address one of the challenges faced by the pig industry of improving resilience without compromising productivity.

## Methods

### Animal material

A total of 432 commercial Duroc pigs (215 females and 217 males) were employed in the present work. The pigs originated from six batches (72 ± 1 animals per batch) and were part of 134 litters produced by 132 sows and 22 boars (all active boars in the commercial Batallé Duroc population). Within each litter, two to four piglets were chosen for this work, balancing gender when possible [14]. Pigs were raised in the same farm until the fattening period where they were distributed in two different farms and fed ad libitum with a commercial cereal-based diet. All animals were healthy and showed no signs of infection. Considering the high cost of slaughtering pigs at 60 days and the ethical issues related to animal welfare, only blood samples were collected at 60 ± 8 days of age via the jugular vein [14]. In addition, the blood FA composition in young animals may be useful to predict production traits at slaughter. Animals were slaughtered at 181–228 days of age in a commercial abattoir, and adipose tissue (backfat), liver, and *gluteus medius* muscle samples were collected immediately after slaughter using RNAlater (ThermoFisher, Spain) and snap-frozen at − 80 °C for further analysis.

The FAs were identified and quantified by gas chromatography (GC) of methyl esters (FAMEs). Swine plasma FAs were analyzed at the IRBLleida Lipidomics Core Facility – PLICAT following previously described methods [15]. In brief, 10 μl of plasma were used for FAMEs extraction and separation was performed with a DBWAX capillary column (30 m × 0.25 mm × 0.20 μm) in a GC System 7890A with a Series Injector 7683B and an FID detector (Agilent Technologies, Barcelona, Spain). Identification of FAMEs was by comparison with authentic standards (Larodan Fine Chemicals, Malmö, Sweden). FA composition from liver, backfat (taken between the third and fourth last ribs), and *gluteous medious* muscle samples were analyzed in the NUTRICAL-UCM laboratory. Samples were extracted and methylated using the procedure described by Segura and Lopez-Bote [16]. FAMEs were identified and quantified by GC (6890 Hewlett Packard, Avondale, PA, USA), using a capillary column (HP- Innowax, 30 m × 0.32 mm id and 0.25 μm cross-linked polyethylene glycol) (Agilent Technologies GmbH, Wald-bronn, Germany) and standards (Sigma-Aldrich, Tres Cantos, Madrid, Spain). FA concentration was expressed as percentage of the total FAs. The percentages of N3, omega-6 (N6), omega-6/omega-3 (N6/N3), saturated FAs (SFAs), monounsaturated FAs (MUFAs), and PUFAs were obtained as the sum of individual FAs. The ratio between individual FA was calculated as product/precursor. Partial least square (PLS) correlations between FA profiles in plasma, liver, adipose tissue, and muscle were calculated using the *block.plsda* function of mixOmics [17] package in R.

### Single‑nucleotide polymorphism (SNP) genotyping

The GeneSeek Genomic Profiler (GGP) Porcine HD Array (Illumina, San Diego, CA) was used to genotype all animals for 63,072 SNPs. Genotypes at the whole genome level were imputed using whole-genome sequence (WGS) data from 1602 pigs from over 100 pig breeds from the multi-breed Pig Genomics Reference Panel (PGRP v1) from PigGTEx [18]. A total of 42,523,218 biallelic SNPs were obtained after imputation with Beagle (v5.2) [19], with an average imputation accuracy of 0.904. SNPs with a minor allele frequency (MAF) lower than 5% were removed using PLINK v.1.90b3.42 software [20], retaining 9,751,141 SNPs for subsequent analysis.

### Genome‑wide association study (GWAS)

GWAS was performed using the 9,751,141 imputed and filtered SNPs for the FA traits from the four tissues with the fastGWA tool from GCTA v.1.94.1 software [21] using the following mixed model:

$${\text{y}}_{\text{ijk}} = {\text{sex}}_{\text{j}}+{\text{batch}}_{\text{k }}+{\beta}_{\text{ci}}+{\text{u}}_{\text{i}}+{\text{s}}_{\text{li}}{{\text{a}}}_{1}+{\text{e}}_{\text{ijk}}$$

where ${\text{y}}_{\text{ijk}}$ indicates the phenotypic trait (raw data and log-transformed) of the i^th^ individual of sex j in the k^th^ batch; ${\text{sex}}_{\text{j}}$ corresponds to the j^th^ sex effect (2 levels); ${\text{batch}}_{\text{k}}$ corresponds to the k^th^ batch effect (6 levels); $\beta$ is a covariate coefficient, with c being the covariate used, i.e. carcass weight for FA traits in liver, muscle, and blood, and backfat thickness for FA traits in backfat; u_i_ is the infinitesimal genetic effect of individual i, with vector $\mathbf{u}\sim \text{N }\left(0,\mathbf{G}{\upsigma }^{2}\text{u}\right)$, where **G** is the sparse genomic relationship matrix (GRM) that was created from the full GRM using the filtered SNPs and default options described in [21]; ${\upsigma }^{2}\text{u}$ is the additive genetic variance; ${\text{s}}_{\text{li}}$ is the genotype code (0, 1, 2) for the l^th^ SNP; ${\text{a}}_{1}$ is the allele substitution effect of the SNP on the trait under the study; and ${\text{e}}_{\text{ijk}}$ is the residual term.

To quantify statistical significance of the associations at the genome-wide level, the false discovery rate (FDR) was calculated for each trait according to Benjamini and Hochberg [22] to correct for multiple testing. The cut-off threshold for considering a SNP as significant was set at FDR ≤ 0.01. Q–Q plots of the p-value distribution were created using the CMplot R package, accessible at: https://github.com/YinLiLin/CMplot.

### QTL definition, gene annotation, and functional prediction

The QTL intervals that contained at least 50 significant consecutive SNPs and with distances between SNPs less than 1 Mb were selected and 0.5 Mb was added to each side of the interval for gene annotation. QTLs with overlapping regions or that were separated by less than 10 Mb were merged.

The BioMart tool from the Ensembl project (Release 110) was used to retrieve gene annotation using the *Scrofa11.1* reference assembly. In addition, functional predictions of the significant SNPs were performed with the Variant Effect Predictor tool [23] from the Ensembl project (Release 110).

## Results

### Descriptive statistics of fatty acid composition

The statistical description of the main FA traits is in Table 1. The relative abundances of 31 individual FAs were measured and 25 FA indexes or ratios were calculated from these (see Additional file 1: Table S1). The FA composition differed between tissues, with PUFA more abundant in blood, SFA in liver, and MUFA in muscle and backfat. For individual FA, blood had a higher content of C18:2*n*-6 (33.0%), C16:0 (20.4%), C18:0 (15.0%) and C18:1*n*-9 (14.8%), while liver had a higher content of C18:0 (30.0%), C16:0 (22.2%), C18:1*n*-9 (21.3%), and C18:2*n*-6 (11.2%). Conversely, muscle and backfat had more similar FA compositions, with a higher content of C18:1*n*-9 (41.1% in backfat, 38.8% in muscle), C16:0 (24.3% in backfat, 23.0% in muscle), C18:0 (13.3% in backfat, 11.6% in muscle) and 18:2*n*-6 (11.5% in backfat, 12.4% in muscle), compared to other FAs in liver and blood. In addition, the N6/N3 ratio was higher in blood (33.0%) in comparison to other tissues, with C18:2*n*-6 being the predominant N6 FA (33.0%), followed by C20:4*n*-6 (6.4%).

**Table 1:** Descriptive statistics fatty acid (FA) composition traits and metabolic indices in liver, muscle, backfat, and plasma of Duroc pigs

Trait	Name	Blood			Liver			Backfat			Muscle		
		mean	SD	CV	mean	SD	CV	mean	SD	CV	mean	SD	CV
C14:0	Myristic acid	1.13	0.72	0.64	0.61	0.26	0.43	1.4	0.13	0.09	1.59	0.24	0.15
C16:0	Palmitic acid	20.38	1.44	0.07	22.19	2.08	0.09	24.28	1.18	0.05	23	2.97	0.13
C18:0	Stearic acid	15.01	1.49	0.1	29.96	5.25	0.18	13.32	1.54	0.12	11.56	1.5	0.13
C20:0	Arachidic acid	–	–	–	0.24	0.11	0.45	0.2	0.03	0.16	0.15	0.05	0.34
C16:1*n*-7	Palmitoleic acid	0.59	0.12	0.2	1.1	0.4	0.36	2.01	0.31	0.15	2.74	0.57	0.21
C18:1*n*-7	Vaccenic acid	1.89	0.34	0.18	2.24	0.31	0.14	2.53	0.34	0.13	3.67	0.7	0.19
C16:1*n*-9	7-Hexadecenoic acid	0.66	0.17	0.26	0.52	0.15	0.29	0.23	0.07	0.31	0.1	0.08	0.79
C18:1*n*-9	Oleic acid	14.76	1.86	0.13	21.31	3.99	0.19	41.09	2.29	0.06	38.79	4.84	0.12
C20:1*n*-9	Gondoic acid	0.27	0.11	0.4	0.26	0.05	0.21	0.88	0.11	0.12	0.6	0.12	0.21
C24:1*n*-9	Nervonic acid	0.54	0.15	0.27	0.43	0.22	0.5	0.01	0.01	0.5	0.08	0.08	1
C18:3*n*-3	Alpha-Linolenic acid	0.46	0.19	0.41	0.25	0.12	0.47	0.68	0.09	0.13	0.41	0.07	0.18
C20:3*n*-3	Eicosatrienoic acid	–	–	–	–	–	–	0.12	0.01	0.12	0.07	0.02	0.32
C20:5*n*-3	Eicosapentaenoic acid	0.48	0.13	0.27	0.3	0.32	1.05	0.02	0.01	0.54	0.07	0.06	0.85
C22:6*n*-3	Docosahexaenoic acid	1.07	0.25	0.24	0.44	0.24	0.56	0.02	0.01	0.43	0.1	0.08	0.82
C18:2*n*-6	Linoleic acid	32.97	3.47	0.11	11.2	2.39	0.21	11.47	1.64	0.14	12.39	4.69	0.38
C18:3*n*-6	Gamma linolenic acid	–	–	–	0.11	0.07	0.65	0.02	0.01	0.34	0.07	0.05	0.65
C20:2*n*-6	Eicosadienoic acid	0.29	0.12	0.39	0.39	0.08	0.22	0.6	0.07	0.12	0.42	0.08	0.2
C20:3*n*-6	Dihomo-γ-linolenic acid	0.46	0.15	0.32	0.36	0.16	0.45	0.08	0.01	0.18	0.35	0.23	0.67
C20:4*n*-6	Arachidonic acid	6.4	1.17	0.18	5.82	2.26	0.39	0.16	0.03	0.17	2.49	1.84	0.74
C22:4*n*-6	Adrenic acid	–	–	–	0.4	0.22	0.55	0.11	0.02	0.23	0.32	0.21	0.66
Fatty acid group													
SFA	Saturated fatty acids	37.55	2.47	0.07	53.96	5.25	0.1	39.48	2.57	0.07	36.51	3.31	0.09
MUFA	Monounsaturated fatty acids	19.89	2.42	0.12	26.26	4.58	0.17	47.11	2.72	0.06	46.45	4.92	0.11
PUFA	Polyunsaturated fatty acids	42.56	2.93	0.07	19.78	4.61	0.23	13.41	1.8	0.13	17.03	7.33	0.43
N3	Omega 3 fatty acids	2.01	0.35	0.18	1.51	0.49	0.33	0.97	0.11	0.12	1	0.39	0.38
N6	Omega 6 fatty acids	40.55	2.96	0.07	17.91	4.38	0.24	12.35	1.71	0.14	15.68	6.75	0.43
N6/N3		20.86	4.23	0.2	12.77	3.81	0.3	12.74	1.36	0.11	15.49	2.16	0.14
C16:0/C14:0		22.94	10.85	0.47	42.11	15.25	0.36	17.49	1.23	0.07	14.72	2.62	0.18
C16:1*n*-9/C16:0		0.03	0.01	0.27	0.02	0.01	0.26	0.01	0	0.35	0	0	0.93
C16:1*n*-7/C16:0		0.03	0.01	0.2	0.05	0.02	0.31	0.08	0.01	0.16	0.12	0.02	0.17
C18:0/C16:0		0.74	0.09	0.12	1.37	0.3	0.22	0.55	0.05	0.09	0.51	0.09	0.18
C18:1*n*-7/C16:1*n*-7		3.28	0.73	0.22	2.25	0.75	0.34	1.27	0.15	0.11	1.42	0.47	0.33
C18:1*n*-9/C18:0		1	0.18	0.18	0.76	0.31	0.41	3.14	0.48	0.15	3.41	0.67	0.2
C18:3*n*-6/C18:2*n*-6		–	–	–	0.01	0.01	0.54	0	0	0.35	0.01	0	0.36
C18:4*n*-3/C18:3*n*-3		–	–	–	0.44	0.36	0.82	0.1	0.02	0.21	0.16	0.04	0.26
C20:4*n*-6/C20:3*n*-6		15.3	5.82	0.38	17.63	6.36	0.36	1.99	0.33	0.17	6.95	1.36	0.2
C20:0/C18:0		–	–	–	0.01	0	0.44	0.01	0	0.12	0.01	0.01	0.4
C20:1*n*-9/C18:1*n*-9		0.02	0.01	0.52	0.01	0	0.3	0.02	0	0.12	0.02	0	0.16
C20:2*n*-6/C18:2*n*-6		0.01	0	0.42	0.04	0.01	0.16	0.05	0.01	0.1	0.04	0.01	0.21
C20:3*n*-6/C18:3*n*-6		–	–	–	4.32	2.88	0.67	3.94	1.25	0.32	5.2	1.52	0.29

### Genome-wide significant QTLs in blood, liver, backfat and muscle

**Fig. 1:**
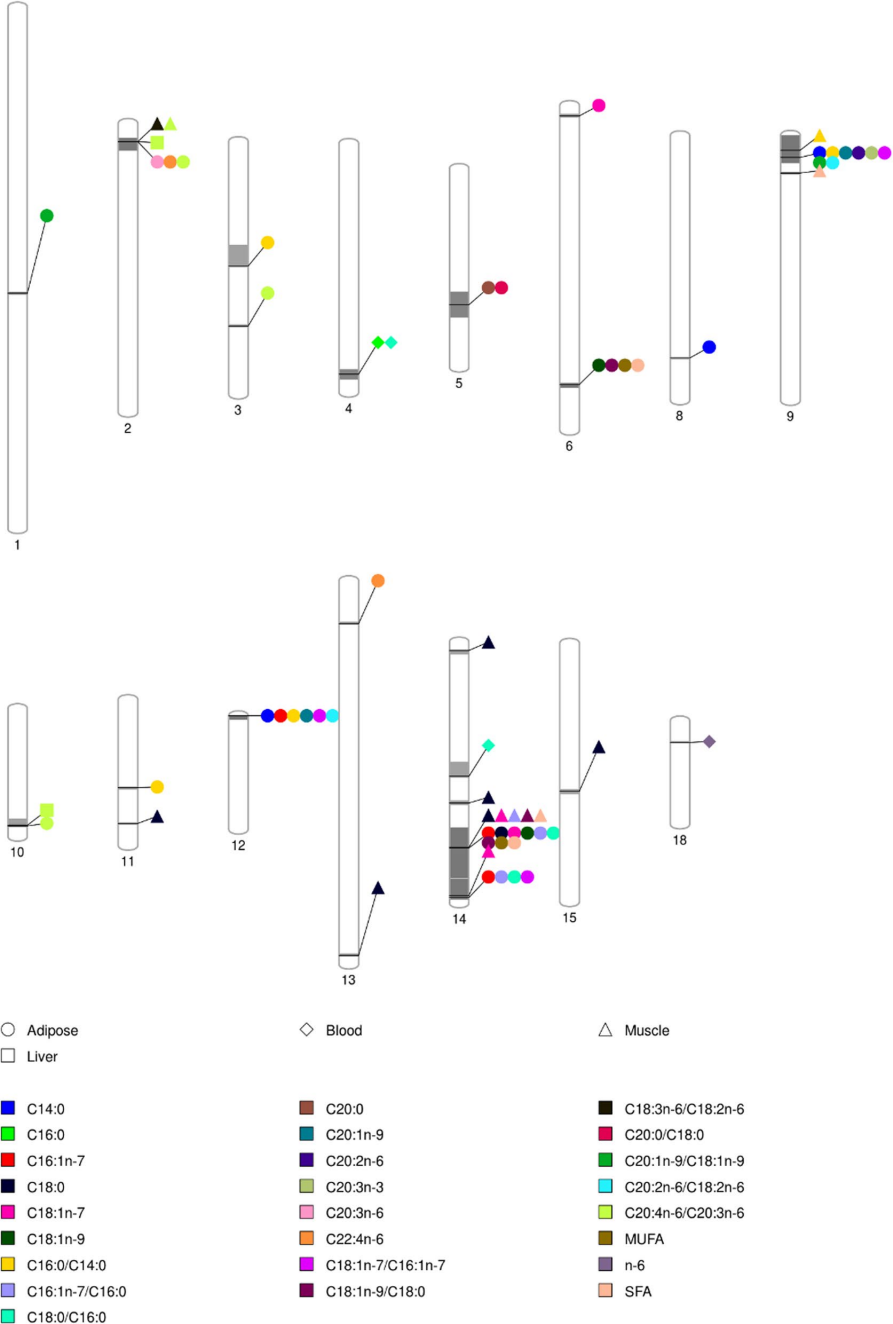
Pig chromosomal regions associated with fatty acid composition in blood, liver, adipose tissue, and muscle. Shapes indicate tissues and colors indicate phenotypic traits

A summary of the significant genomic regions and associated traits is in Fig. 1 and Table 2. For blood, three significant QTLs for three FA traits were identified, on pig chromosomes SSC4, SSC14, and SSC18. For liver FAs, only the ratio of C20:4*n*-6/C20:3*n*-6 had significant QTLs, located on SSC2 and SSC10. Backfat and muscle had more QTLs identified. Fifteen QTLs, on 12 chromosomes, were identified for 22 FA traits in backfat and ten QTLs, on six chromosomes, were detected for eight FA traits in muscle. A total of 108,301 significantly associated SNPs were identified, 10,971 in blood, 9199 in liver, 66,620 in backfat, and 21,511 in muscle. The most significant associated SNPs and their predicted consequences are summarized in Additional file 2: Table S2 and Additional file 3: Table S3. In addition, Manhattan plots and Q–Q plots for the most significant traits for each region are in Additional file 4 Figure S1.

**Table 2:** Genomic regions associated with fatty acid composition traits across four tissues

Tissue	QTL	Chr	Start (bp)	End (bp)	No SNPs	Top SNP	P-value	FDR	Candidate Gene	Trait
Blood	Region B1	4	118,919,374	123,945,538	9413	rs336174148	3.49E−10	0.000285	*ABCD3*, *SLC44A3*	C16:0*; C18:0/C16:0
Blood	Region B2	14	65,308,168	73,742,077	1503	rs325107907	1.26E−06	0.001987	*ARID5B*	C18:0/C16:0
Blood	Region B3	18	10,937,127	12,120,906	55	rs344737196	1.68E−08	0.005637	*CREB3L2*, *AKR1D1*	N6
Liver	Region L1	2	7,563,025	14,268,504	8887	rs344582470	4.55E−18	4.07E−12	*FADS1*, *FADS2*, *FADS3*, *BEST1*, *DAGLA*	C20:4*n*-6/C20:3*n*-6
Liver	Region L2	10	62,589,655	63,879,801	312	rs326143247	3.26E−07	0.000598	*ECHDC3*, *GATA3*	C20:4*n*-6/C20:3*n*-6
Adipose	Region A1	1	149,886,913	151,144,913	309	rs786957380	2.99E−06	0.005213	–	C20:1*n*-9/C18:1*n*-9
Adipose	Region A2	2	7,563,025	14,261,411	9029	rs333815987	4.28E−21	1.62E−14	*FADS1*, *FADS2*, *FADS3*, *BEST1*, *DAGLA*	C20:3*n*-6; C22:4*n*-6; C20:4*n*-6/C20:3*n*-6*
Adipose	Region A3	3	54,581,209	66,007,388	238	rs325400369	1.01E−06	0.001134	*FABP1*	C16:0/C14:0
Adipose	Region A4	3	96,359,613	97,705,761	72	rs324894318	2.94E−06	0.003962	*LRPPRC*, *ABCG5*, *ABCG8*	C20:4*n-*6/C20:3*n*-6
Adipose	Region A5	5	64,835,240	78,318,485	18,530	rs321190930	3.06E−22	1.50E−17	*SLC2A13*, *ADIPOR2*	C20:0; C20:0/C18:0*
Adipose	Region A6	6	4,566,421	6,186,800	150	rs336208372	2.13E−06	0.003626	*CDH13*, *MLYCD*	C18:1*n*-7
Adipose	Region A7	6	146,051,560	148,355,009	457	rs331853003	4.48E−08	0.00013	*LEPR*, *ROR1*	C18:1*n*-9*; C18:1*n*-9/C18:0; MUFA; SFA
Adipose	Region A8	8	116,426,190	117,518,311	74	rs342642240	8.37E−07	0.002866	*CXXC4*	C14:0
Adipose	Region A9	9	0	14,548,445	14,985	rs325219947	2.99E−15	2.40E−09	*GDPD5*, *DGAT2*, *PAK1*, *MOGAT2*, *UCP2*, *UCP3*	C14:0; C20:1*n*-9; C20:2*n*-6; C20:3n3; C16:0/C14:0*; C18:1*n*-7/C16:1*n*-7; C20:1*n*-9/C18:1*n*-9; C20:2*n*-6/C18:2*n*-6
Adipose	Region A10	10	59,635,643	64,344,350	325	rs699684228	6.62E−07	0.001097	*ECHDC3*, *GATA3*	C20:4*n*-6/C20:3*n*-6
Adipose	Region A11	11	47,166,152	48,653,064	59	rs330006520	9.97E−07	0.001119	*TBC1D4*	C16:0/C14:0
Adipose	Region A12	12	0	1,905,028	4714	rs691598356	4.48E−10	6.62E−06	*FASN*, *PCYT2*, *DCXR*	C14:0*; C16:1*n*-7; C20:1*n*-9; C16:0/C14:0; C18:1*n*-7/C16:1*n*-7; C20:2*n*-6/C18:2*n*-6
Adipose	Region A13	13	22,443,412	23,866,356	68	rs328210301	5.32E−09	0.000436	*ACAA1*, *PLCD1*	C22:4*n*-6
Adipose	Region A14	14	103,811,979	115,637,075	17,016	rs320692657	3.40E−13	1.89E−08	*PAX2*, *SCD*, *ELOVL3*, *FFAR4*	C16:1*n*-7; C18:0; C18:1*n*-7; C18:1*n-*9; C16:1*n*-7/C16:0; C18:0/C16:0; C18:1*n*-9/C18:0*; SFA; MUFA
Adipose	Region A15	14	129,014,157	139,724,693	594	rs343554276	4.77E−07	0.000962	*ACADSB*	C16:1*n*-7; C16:1*n*-7/C16:0*; C18:0/C16:0; C18:1*n*-7/C16:1*n*-7
Muscle	Region M1	2	7,884,782	13,736,647	2747	rs319353021	2.99E−14	7.38E−08	*FADS1*, *FADS2*, *FADS3*, *BEST1*, *DAGLA*	C18:3*n*-6/C18:2*n*-6; C20:4*n*-6/C20:3*n*-6*
Muscle	Region M2	9	5,547,024	8,536,688	2069	rs704020821	5.13E−08	0.004406	*UCP2*, *UCP3*	C16:0/C14:0
Muscle	Region M3	9	19,221,645	20,665,362	148	rs338705619	2.09E−07	0.002361	*ME3*	SFA
Muscle	Region M4	11	66,625,592	67,719,689	161	rs321703705	1.15E−06	0.001304	*STK24*	C18:0
Muscle	Region M5	13	202,691,429	203,957,195	63	rs3472333533	2.78E−06	0.002808	–	C18:0
Muscle	Region M6	14	4,173,825	6,576,464	747	rs343455499	4.49E−07	0.000631	–	C18:0
Muscle	Region M7	14	85,889,681	88,383,396	383	rs3471900337	1.25E−07	0.000178	*GLUD1*	C18:0
Muscle	Region M8	14	100,912,624	128,141,538	14,359	rs709198288	1.27E−19	1.01E−13	*ACSL5, SCD*, *ELOVL3*, *FFAR4*, *GOT1*, *PNLIP*, *PNLIPRP1*, *PNLIPRP2*, *PNLIPRP3*	C18:0*; C18:1*n*-7; C16:1*n*-7/C16:0; C18:1*n*-9/C18:0; SFA
Muscle	Region M9	14	137,219,445	138,615,860	212	rs321862224	2.44E−10	0.000112	–	C18:1*n*-7
Muscle	Region M10	15	79,303,831	81,706,260	622	rs706060527	5.00E−06	0.004059	–	C18:0

* indicates the most significant trait

### Candidate genes for significant QTLs for FA in blood

Region B1 (Table 2). The 118.92–123.95 Mb region on SSC4 was associated with the content of C16:0 (palmitic acid) and with the C18:0/C16:0 ratio. This region was found only in blood. The intergenic SNPs rs336174148 and rs345078893 (*p*-value = 3.49E−10) were the top significant associated SNPs for C16:0. In this region, the *ATP Binding Cassette Subfamily D Member 3* (*ABCD3*) from the superfamily of ATP-binding cassette (ABC) transporters was the candidate gene annotated. The *ABCD3* gene is involved in the transport of various lipids, including long chain fatty acids (LCFAs) and very long chain fatty acids (VLCFAs), and specifically transports dicarboxylic acids and branched-chain FAs [24]. The *SLC44A3* gene, a member of the solute carrier family of membrane transporters, was also identified as a candidate gene. This gene has been proposed to be involved in choline transport [25].

Region B2. The 65.31–73.74 Mb region on SSC14 was associated with the C18:0/C16:0 ratio. The most significant SNP in this region was rs325107907 (*p*-value = 1.26E−06), which is located in an intron of the *COL13A1* gene. The candidate gene *AT-rich interaction domain 5B* (*ARID5B*) is a member of the AT-rich interaction domain (ARID) family. Suppression of *ARID5B* expression in mice significantly inhibited adipogenesis, reducing white fat mass [26].

Region B3. The 10.94–12.12 Mb region on SSC18 was associated with N6 content in blood. The nine top significant SNPs (*p*-value = 1.68E−08) for this region were located in an intron of *CREB3L2* (CAMP responsive element binding protein 3 like 2, see Additional file 2: Table S2). Another candidate gene annotated in this region was *Aldo–Keto reductase family 1 member D1* (*AKR1D1*) which is involved in cholesterol metabolism. The *AKR1D1* knockout elevated insulin sensitivity in human hepatic cells and encouraged hepatocyte TAG accumulation by enhancing lipogenesis and decreased oxidation [27].

### Candidate genes for significant QTLs for FA in liver

Region L1. The 7.56–14.27 Mb region of SSC2 was associated with the metabolic ratio C20:4*n*-6/C20:3*n*-6 (D5 activity). The most significant SNP (rs344582470; *p*-value = 4.55E−18) was located in an intron of the *FADS3* gene. In this region, the *FADS* (fatty acid desaturase) gene cluster was annotated, which includes *FADS1*, *FADS2,* and *FADS3*. The *FADS* genes are involved in the synthesis of PUFA, inserting the unsaturated bonds into FA molecules [28]. While *FADS1* and *FADS2*, respectively, encode the rate-limiting enzymes delta-5 (D5) and D6, *FADS3* encodes an enzyme with an unclear specific biological function [29]. In addition, two other candidate genes, *Bestrophin 1* (*BEST1*) and *DAGLα* (*DAGLA,* *Diacylglycerol lipase alpha*), were found in this region. These two genes have been reported to be respectively associated with C20:3*n*-6 (dihomo-γ-linolenic acid, DGLA) levels in red blood cells in human and involved in 2-arachidonoylglycerol (2-AG) synthesis [30].

Region L2. The second region associated with the metabolic ratio C20:4*n*-6/C20:3*n*-6 was located at 62.59–63.88 Mb of SSC10. The most significant SNP was rs326143247 (*p*-value = 3.26E−07), which was located in an intergenic region. As described above, the ratio of C20:4*n*-6/C20:3*n*-6 reflects the activity of the D5 enzyme that is encoded by *FADS1*. It is worth noting that the transcription factor GATA-binding protein 3 (GATA3) was also located in this region. The GATA3 belongs to the GATA family of transcription factors and inhibits key lipogenic regulator PPARγ2 by binding to their promoters [31]. In addition, the candidate gene *ECHDC3* (*Enoyl-CoA hydratase domain containing 3*) was located in this region. *ECHDC3* is related to mitochondrial beta-oxidation but with an unclear role [32].

### Candidate genes for significant QTLs for FA in backfat

Region A2. The 7.56–14.26 Mb region on SSC2 was associated with the abundance of DGLA, C22:4*n*-6 (adrenic acid), and metabolic ratio C20:4*n*-6/C20:3*n*-6 in backfat. The most significant SNP was rs333815987 (*p*-value = 4.28E−21) for C20:4*n*-6/C20:3*n*-6, which was located in an intron of the *FADS2* gene.

Region A3. The 54.58–66.01 Mb region of SSC3 was associated with the C16:0/C14:0 ratio. The most significant SNP was rs325400369 (*p*-value = 1.01E−06), which was located in an intergenic region. The candidate gene *fatty acid binding protein 1 (FABP1*, also named *LFABP*) is not only involved in hepatic FA metabolism but also impacts whole-body energy homeostasis [33]. Remarkably, the C16:0/C14:0 ratio QTL overlaps with a cis-eQTL (SSC3: 58.0–62.5 Mb) for *FABP1* mRNA expression in liver [34].

Region A4. The region 96.36–97.71 Mb on SSC3 was associated with the ratio of C20:4*n*-6/C20:3*n*-6. The most significant SNP was rs324894318 (*p*-value = 2.94E−06), which is a synonymous variant of *ZFP36L2* (*ZFP36 ring finger protein like 2*). One of the candidate genes annotated in this region, the *LRPPRC* (*Leucine rich pentatricopeptide repeat-containing*) gene, promotes FA uptake and oxidation and regulates energy metabolism by increasing oxidative phosphorylation or by regulating the activity of ATP synthase [35]. Two additional candidate genes were identified for this region, *ABCG5* and *ABCG8* (ABCG5/G8), which belong to the ABCG subfamily of ABC transporters. ABC transporters can mediate the transmembrane transport of various endogenous molecules powered by hydrolysis of ATP [36]. The natural environments of ABCG5/G8 are the lipid bilayers of the plasma membranes, which are not only used as communication barriers and passive permeation but also have crucial signaling functions by using ABCC family substrates such as AA-derived prostaglandins or leukotrienes [37].

Region A5. The 64.84–78.32 Mb region of SSC5 was associated with the percentage of C20:0 (arachidic acid) and the ratio of C20:0/C18:0. Thirteen SNPs with the same *p*-value (3.06E−22) were the most significant (see Additional file 2: Table S2), all located in intronic or upstream regions of *SLC2A13* (*Solute carrier family 2 member 13*), or in an intergenic region close to *SLC2A13*. The candidate gene *ADIPOR2* is one of the adiponectin receptors and is well known to be associated with homeostasis through anti-inflammatory, anti-fibrotic, and antioxidant effects [38]. Another candidate gene found in this region was *ABCD2*, a member of ABC transporters, involved in peroxisomal beta-oxidation.

Region A6. The 4.57–6.19 Mb region of SSC6 was associated with the content of C18:1*n*-7 (vaccenic acid). The most significant SNP rs336208372 (*p*-value = 2.13E−06) was located in an intron of *CDH13* (*Cadherin 13*), which is a member of the cadherin superfamily. In *CDH13* knockout mouse, FA uptake and lipid content decreased in developing adipocytes during adipogenesis [39]. Another candidate gene for this region, *MLYCD* (*Malonyl-CoA decarboxylase*) encodes an enzyme involved in the degradation of malonyl-CoA which is the regulator of cellular FA oxidation and lipid partitioning [40].

Region A7. Another region of SSC6 (146.05–148.36 Mb) was associated with the percentage of C18:1*n*-9 (oleic acid) and with three FA metabolic ratios C18:1*n*-9/C18:0, MUFA, and SFA. The most significant SNP was the intergenic rs331853003 (*p*-value = 4.48E−08) for C18:1*n*-9. The candidate gene *leptin receptor* (*LEPR*) was located in this QTL region. The receptor *ROR1* (*Receptor tyrosine kinase like orphan receptor 1*) was also located in this region. The RNA-Seq analysis of Tanaka et al. [41] revealed that in cultured astrocytes, the *ROR1* expression regulates the FA metabolism-related gene expression mediated by PPARα and promotes the availability of lipid droplets-derived FAs for mitochondrial oxidation.

Region A8. The 116.43–117.52 Mb region of SSC8 was associated with the abundance of C14:0 (myristic acid). The eight top significant associated SNPs (*p*-value = 8.37E−07) found in this region (see Additional file 2: Table S2) were located in the lncRNA ENSSSCG00000057642 sequence. The candidate gene *CXXC finger protein 4* (*CXXC4*), was also annotated in this region and is involved in inhibiting cancer cell growth by regulating the Wnt/β-catenin pathway [42].

Region A9. The 0 – 14.55 Mb region of SSC9 was associated with the abundance of four FAs (C14:0, C20:1*n*-9, C20:2*n*-6, C20:3*n*-3) and with four FA metabolic ratios (C16:0/C14:0, C18:1*n*-7/C16:1*n*-7, C20:1*n*-9/C18:1*n*-9, C20:2*n*-6/C18:2*n*-6). The most significant SNP was rs325219947 (*p*-value = 2.99E−15) for C16:0/C14:0, which was located in intron 1 of the gene *GDPD4* (*Glycerophosphodiester phosphodiesterase domain-containing protein 4*). The candidate gene *glycerophosphodiester phosphodiesterase* (*GDPD5*) was also found in this region. It encodes an enzyme involved in glycerol metabolism and related energy production. Overexpression of *GDPD5* in human cells inhibited cell proliferation and migration and affected lipid metabolism [43]. The candidate gene *diacylglycerol O-acyltransferase 2* (*DGAT2*) was also located in this region. It catalyzes the final step of the TAG synthesis [44, 45]. Another candidate gene found for this region was the *monoacylglycerol O-acyltransferase* (*MOGAT2*) gene. *MOGAT* genes encode the monoacylglycerol acyltransferase (MGAT) isoforms *(MGAT1, MGAT2,* and *MGAT3)*, which catalyze the conversion of monoacylglycerol (MAG) to DAG, the first step of TAG production, followed by the step catalyzed by DAGT2 [46]. The last two candidate genes annotated in this region were *uncoupling protein 2* (*UCP2*) and *UCP3*, which are expressed in muscle and adipose tissue and are capable of uncoupling ATP produced by mitochondrial respiration, thereby affecting the efficiency of energy metabolism by dissipating energy in the form of heat [47].

Region A10. The 59.64–64.34 Mb region of SSC10 was the second region associated with the metabolic ratio of C20:4*n*-6/C20:3*n*-6. The SNPs rs699684228 and rs331489813 were the most significant SNPs (*p*-value = 6.62E−07), which were located in an intergenic region. The candidate genes for this region have already been described for liver region L2.

Region A11. The 47.17–48.65 Mb region of SSC11 was associated with the level of C16:0/C14:0. A total of 37 top significant SNPs (*p*-value = 9.97E−07) were identified in this region (see Additional file 2: Table S2), all located in the first intron of the *TBC1D4* (*TBC1 domain family member 4*) gene. *TBC1D4* encodes a RabGTPase-activating protein (RabGTP). Mice muscle cells with RabGTPs deficiency showed an elevated C16:0 uptake and oxidation [48].

Region A12. The 0–1.91 Mb region of SSC12 was associated with the abundance of three FAs (C14:0, C16:1*n*-7, 20:1*n*-9) and with three FA metabolic ratios (C16:0/C14:0, C18:1*n*-7/C16:1*n*-7, C20:2*n*-6/C18:2*n*-6). The three most significant SNPs (*p*-value = 4.48E−10) for C14:0 were located at the upstream region and exon of lncRNA ENSSSCG00000055585, and in an intergenic region. The *fatty acid synthase gene* (*FASN*) was a relevant candidate gene found in this region, as it is directly involved in de novo synthesis of C16:0 and C14:0 FAs [49]. In addition, *FASN* has a crucial role in membrane structure, protein modification, and location function, and also participates in signaling pathways such as PI3K/AKT/mTOR [50]*.* Other potential candidate genes identified in this region were the *phosphatidylethanolamine cytidyltransferase* (*PCYT2*) and the *dicarbonyl/l-xylulose reductase* (*DCXR*) genes. *PCYT2* is the key enzyme in de novo biosynthesis of phosphatidylethanolamine ethanolamine and diacylglycerol by the CDP-ethanolamine Kennedy pathway [51]. *PCYT2*-deficient mice had higher palmitic acid (C16:0) and stearic acid (C18:0) contents in hepatic phospholipids compared to control mice [52]. The *DCXR* gene is involved in AA metabolism and is associated with C14:0 [53].

Region A13. The 22.44–23.87 Mb region of SSC13 was associated with the abundance of C22:4*n*-6 (adrenic acid). The most significant SNP was rs328210301 (*p*-value = 5.32E−09), which was located in the intron 2 of the *EXOG* gene. The *acetyl-CoA acyltransferase 1* (*ACAA1*) candidate gene in this region encodes an enzyme involved in lipid beta-oxidation, supplying the substrates to the tricarboxylic acid (TCA) cycle [54]. Another lipid metabolism-related candidate gene annotated in this region was *PLCD1* (*Phospholipase C delta 1*). Liu et al. [55] reported that the expression level of *PLCD1* in chicken muscle was negatively correlated with the C18:2*n*-6 content.

Region A14. The 103.81–115.64 Mb region of SSC14 was associated with four FA contents (C16:1*n*-7, C18:0, C18:1*n*-7, C18:1*n*-9) and with five FA metabolic ratios (C16:1*n*-7/C16:0, C18:0/C16:0, C18:1*n*-9/C18:0, SFA, MUFA). The three most significant SNPs (*p*-value = 3.40E−13) for C18:1*n*-9/C18:0 were located between the lncRNA ENSSSCG00000058770 and the transcription factor *PAX2* (Paired box 2). Feng et al. [56] reported that expression of *PAX2* in mouse ovarian surface epithelial cells promotes the use of FAs as an energy source by increasing the expression of enzymes involved in FA metabolism, mitochondrial oxidative phosphorylation pathways, and the components of the mitochondrial electron transfer chains to generate ATP. In addition, the blood ratios of C16:1*n*-7/C16:0 and C18:1*n*-9/C18:0 reflect the activity of the *stearoyl CoA desaturase* (*SCD*) [57]. *SCD*, one of the candidate genes annotated in this region, catalyzes the SFA precursors to synthesize the MUFA, and its activity is positively correlated with MUFA content [58]. In addition to *SCD*, *ELOVL3* (*ELOVL Fatty Acid Elongase 3*) is another widely reported gene related to FA metabolism that was found in this QTL region. *ELOVL3* encodes an enzyme that is involved in the biosynthesis of VLCFAs [59]. The last candidate gene for this region was the *free fatty acid receptor 4* (*FFAR4*) gene, which encodes a G-protein coupled receptor whose endogenous ligands are N3 PUFAs [60, 61].

Region A15. The region at 129.01–139.72 Mb of SSC14 was associated with the percentage of C16:1*n*-7 (palmitoleic acid) and with three FA metabolic ratios (C16:1*n*-7/C16:0, C18:0/C16:0, and C18:1*n*-7/C16:1*n*-7). The most significant SNP was rs343554276 (*p*-value = 4.77E−07) for C16:1*n*-7/C16:0, which was located in an intron of the *MGMT* gene. Another candidate gene in this region, *ACADSB* (*Short/branched-chain acyl-CoA dehydrogenase*), belongs to the acyl-CoA dehydrogenase family, regulates FA oxidation by catalyzing the dehydrogenation of acyl-CoA derivatives, and is positively correlated with the level of TAG in mammary epithelial cells of dairy cattle [62].

### Candidate genes for significant QTLs for FA in muscle

Region M1. The 7.88–13.74 Mb region of SSC2 was associated with the two FA metabolic ratios, C18:3*n*-6/C18:2*n*-6 and C20:4*n*-6/C20:3*n*-6, reflecting the elongase activity of the *FADS2* and *FADS1* genes, respectively. The most significant SNPs for C20:4*n*-6/C20:3*n*-6 were rs319353021, rs318386280, and rs345083985 (both *p*-value = 2.99E−14), which were located between the *INCENP* (*Inner Centromere Protein*) gene and lncRNA ENSSSCG00000062139. Candidate genes in this region were described above, with *FADS1* and *FADS2* as the most relevant genes, as they are directly involved in the desaturation process of these FA ratios.

Region M2. The 5.55–8.54 Mb region of SSC9 was associated with the C16:0/C14:0 ratio. The most significant SNP for C16:0/C14:0 was rs704020821 (*p*-value = 5.13E−08), which was located in an intron of the *FAM168A* (*Family with sequence similarity 168 member A*) gene. Other candidate genes for this region, *UCP2* and *UCP3,* were already described for the backfat region A9.

Region M3. Another region (19.22–20.67 Mb) on SSC9 was associated with SFA. The rs338705619 was the most significant SNP (*p*-value = 2.09E−07) and was located in the intron 3 of the *PICALM* (*Phosphatidylinositol binding clathrin assembly protein*) gene. The candidate gene annotated in this region was *ME3* (*Malic enzyme 3*), which is involved in the FA synthesis (FAS) and pyruvate metabolism pathway [63].

Region M7. The 85.89 – 88.38 Mb region located on SSC14 was associated with the abundance of C18:0. The SNP rs3471900337 (*p*-value = 1.25E−07) was the most significant variant and was located in an intergenic region. One candidate gene, *glutamate dehydrogenase 1* (*GLUD1*), was found in this region. *GLUD1* is involved in energy metabolism by encoding the enzyme that catalyzes glutamate to α-ketoglutarate, which is the first step in generating citrate and acts as a carbon source to fuel FAS [64, 65].

Region M8. The region at 100.91–128.14 Mb on SSC14 was associated with the abundance of two FAs (C18:0 and C18:1*n*-7) and with three FA metabolic ratios (C16:1*n*-7/C16:0, C18:1*n*-9/C18:0, SFA). The most significant SNP for this QTL was rs709198288 (*p*-value = 1.27E−19) for C18:0, which was located in an intron of *PKD2L1* (*Polycystin 2 like 1*). The most relevant candidate genes (*SCD*, *ELOVL3*, and *FFAR4*) in this region were already described for adipose region 14. However, additional candidate genes for this region included *GOT1* (*Glutamic-oxaloacetic transaminase 1*), which is involved in cytoplasmic aspartate synthesis [66]. The *GOT1* gene is also involved in a reversible reaction that catalyzes the production of oxaloacetate (OAA) from aspartate, which is transferred from the mitochondria to the cytoplasm. Subsequently, the generated OAA is converted to phosphoenolpyruvate, which is utilized for gluconeogenesis [67]. The *ACSL5* (*Acyl-CoA synthetase long chain family member 5*) found in this region prefers LCFA as a substrate for the formation of acyl-CoAs, which are then used to synthesize complex lipids or enter mitochondria for beta-oxidation [68]. Another candidate gene in this region is the *pancreatic triglyceride lipase* (*PNLIP*) gene, which encodes the lipolytic enzymes that mediate the digestion and absorption of dietary fat [69, 70]. During pancreatitis in humans, PNLIP content increased in adipose tissue and entered visceral adipocytes, inhibiting mitochondrial complexes I and V through the production of long-chain NEFA by hydrolyzing adipose TAG [71]. Related family member genes were also identified in this region, including *PNLIPRP1*, *PNLIPRP2*, and *PNLIPRP3*.

Region M9. The 137.22–138.62 Mb region on SSC14 was associated with the abundance of C18:1*n*-7 (vaccenic acid). The most significant SNPs (*p*-value = 2.44E−10) were located in an intergenic region. No candidate genes were identified in this region.

Regions M4, M5, M6, and M10 were associated with the abundance of muscle C18:0 (stearic acid) and distributed on different chromosomes. For the M4 region on SSC11 at 66.63–67.72 Mb, the SNPs rs321703705 and rs329478531 were the most significant (*p*-value = 1.15E−06) and were located in an intron of the *FARP1* (*ARH/RhoGEF and pleckstrin domain protein 1*) gene. The candidate gene *STK24* (Serine/Threonine kinase 24) was identified in this region. *STK24*–deficient mice fed a high-fat diet had induced metabolic disorders and insulin insensitivity [72]. The M5 region was located on SSC13 at 202.69–203.96 Mb. The most significant SNP in this region was rs3472333533 (*p*-value = 2.78E−06), which was located in an intron of *PCP4* (*Purkinje cell protein 4*). The M6 region was located on SSC14 at 4.17–6.58 Mb. The six most-significant SNPs (*p*-value = 4.49E−07) were located in an intergenic region. The M10 region associated with the abundance of C18:0 was on SSC15 at 79.30–81.71 Mb. The most significant SNP was rs706060527 (*p*-value = 5.00E−06), which was located in an intron of the novel gene *WIPF1* (*WAS/WASL interacting protein family member 1*). No candidate genes were found in these three genomic regions.

### Comparison between QTL across tissues

Several genomic regions were found to be associated with FA composition and ratios across tissues. The first common region was located on SSC2 and was associated with the C20:4*n*-6/C20:3*n*-6 ratio in backfat (7.56–14.26 Mb), liver (7.56–14.27 Mb), and muscle (7.88–13.74 Mb). In backfat and liver, the most significant SNPs were located in the introns of *FADS2* (SSC2: 9,672,755) and *FADS3* (SSC2: 9,622,157) respectively. And in muscle, the most significant SNPs were located in an intergenic region (around SSC2: 9,465,204), which was 0.27 Mb upstream from *FADS1*. In muscle, the common QTL was not only associated with the C20:4*n*-6/C20:3*n*-6 ratio but also with the C18:3*n*-6/C18:2*n*-6 ratio. The most significant SNP for C18:3*n*-6/C18:2*n*-6 ratio was located in an intron of *SYT7*.

The second common genomic region, on SSC10 for backfat (59.64–64.34 Mb) and liver (62.59–63.88 Mb), was associated with the C20:4*n*-6/C20:3*n*-6 ratio.

On SSC9, a common region associated with the C16:0/C14:0 ratio was found for backfat (0–14.55 Mb) and muscle (5.55–8.54 Mb). For backfat, this QTL was also related to four FAs (C14:0, C20:1*n*-9, C20:2*n*-6, and C20:3*n*-3) and three ratios (C18:1*n*-7/C16:1*n*-7, C20:1*n*-9/C18:1*n*-9, and C20:2*n*-6/C18:2*n*-6).

On SSC14, a common region for backfat (103.81–115.64 Mb) and muscle (100.91–128.14 Mb) was associated with multiple FA traits (C18:0, C18:1*n*-7, C16:1*n*-7/C16:0, C18:1*n*-9/C18:0, SFA). For muscle, this QTL was also associated with two FAs (C16:1*n*-7 and C18:1*n*-9) and two ratios (C18:0/C16:0 and MUFA).

The 137.22–138.62 Mb region on SSC14 was associated with the abundance of C18:1*n*-7 in muscle. This region was also identified in backfat (Region A15: 129.01–139.72 Mb) but associated with different FA traits.

## Discussion

### GWAS for fatty acid traits identified relevant candidate genes for fatty acid metabolism

Several publications have reported on the genetic architecture of FA composition in backfat and in the *longissimus dorsi* muscle in pigs. Previous studies showed moderate to high heritabilities for FA composition in muscle and adipose tissues in pigs [73–75]. In Large White and Landrace pigs, the estimated heritabilities for FA composition in backfat and muscle were in the range between 0.40 and 0.70 [74]. Conversely, a study in Iberian pigs reported heritabilities ranging from 0.28 to 0.67 for FA composition of subcutaneous fat and *longissimus thoracis* muscle [75].

Previous studies have also identified significant QTLs and candidate genes in muscle and fat tissues of pigs [76–80]. However, there is less insight into the genomic regions that control FA composition in liver and blood. The present work compared the FA profile and its genetic architecture in four metabolic tissues obtained from the same pigs.

In our study, we identified genes and SNPs associated with FA metabolism across blood, liver, adipose tissue, and muscle. The findings clarified the mechanisms that determine FA profiles in different tissues of the pig and may contribute to selection programs aimed at improving pig health and energy metabolism. The tissues with the highest number of QTLs identified were backfat and muscle, followed by blood and liver. In addition, backfat was the tissue with more pleiotropic QTLs, i.e. QTLs that affect more than six traits**.**

Remarkably**,** the QTL at 7.88–13.74 Mb on SSC2 that was common to backfat, liver, and muscle was associated with D5 activity (C20:4*n*-6/C20:3*n*-6). The same genomic region had been previously reported for C20:4*n*-6/C20:3*n*-6 in muscle of Erhualian pigs [76]. The D5 enzyme introduces the double bonds to the D5 position of DGLA and AA [81]. These two FAs, C20:4*n*-6 and C20:3*n*-6, are the precursors of the eicosanoids, which are the FA metabolites that regulate physiological and pathological functions, including homeostasis, host defense, and inflammation [82]. Therefore, *FADS1*, a key gene in PUFA metabolism, encodes the rate-limiting enzyme D5; the balance of C20:4*n*-6 and C20:3*n*-6 levels and the *FADS1* gene activity is important in lipid and inflammation metabolism.

In addition, *GATA3* was annotated in the liver and backfat QTL for D5 activity on SSC10. This transcription factor has been predicted by the JASPAR database [83] to bind to the promoter region of *FADS1* and was proved to up-regulate *FADS2* expression in liver of rainbow trout [84]. Further analyses are warranted to verify the transcriptional regulation of *FADS1* by *GATA3*. Furthermore, in muscle, the common QTL identified on SSC2 was also associated with the ratio of C18:3*n*-6/C18:2*n*-6, which reflects *FASD2* activity.

Two neighboring regions on SSC8 and one region on SSC12 were found to be both associated with C14:0 content of backfat in this study (Region A8, Region A12) and QTL for C14:0 levels that our group previously identified in backfat of a Landrace backcross (SSC8: 108.40–116.41 Mb, SSC12: 0–1.91 Mb, [85]). The *FASN* gene, which is involved in regulating C14:0 synthesis, was located in the QTL on SSC12 that was common across these studies. In addition, a common region on SSC9 at 19.22–20.67 Mb was also found in the current study and our previous work [85] and was associated with SFA content and C20:1*n*-9/C20:0.

Numerous studies in different pig populations have identified FA-associated regions on SSC14. Remarkably, the region on SSC14 at 100.91–128.14 Mb that was associated with the C18:1*n*-9/C18:0 ratio was shared across tissues and breeds [76, 78, 85]. The candidate gene *SCD* is involved in the synthesis of C18:1*n*-9 from C18:0. *SCD* introduces a double bond at position delta-9 and converts SFA into MUFA, a mediator of signal transduction, cell differentiation, and metabolic homeostasis [86]. Therefore, the balance of *SCD* activity is crucial to health, and its deficiency induces metabolic disease, including adiposity decrease, lipid oxidation, and insulin sensitivity increase [87].

In addition to QTLs that were common across tissues and traits, some tissue-specific regions were also identified. For blood, the QTL (118.92–123.95 Mb) on SSC4 was associated with C16:0 and C18:0/C16:0 ratio content. C16:0 is one of the specific substrates of the candidate gene *ABCD3*, which transports mitochondrial FAs substrates into peroxisome to perform beta-oxidation [88]. The FA C20:0 in backfat and the ratio of its precursors (C20:0/C18:0) were found to be uniquely associated with the QTL on SSC5. The promising candidate gene *ABCD2* found in this region participates in the metabolism of VLCFA in peroxisomes. The peroxisomal ABC transporter is not limited to the catabolic functions of the peroxisome but is implicated in a variety of metabolic functions, including lipid synthesis and degradation, cell signaling, inflammation control, and redox homeostasis [24]. However, experiments are needed to prove the association between ABC transporters and FA metabolism.

### Deducing fatty acid metabolic pathways from GWAS

**Fig. 2:**
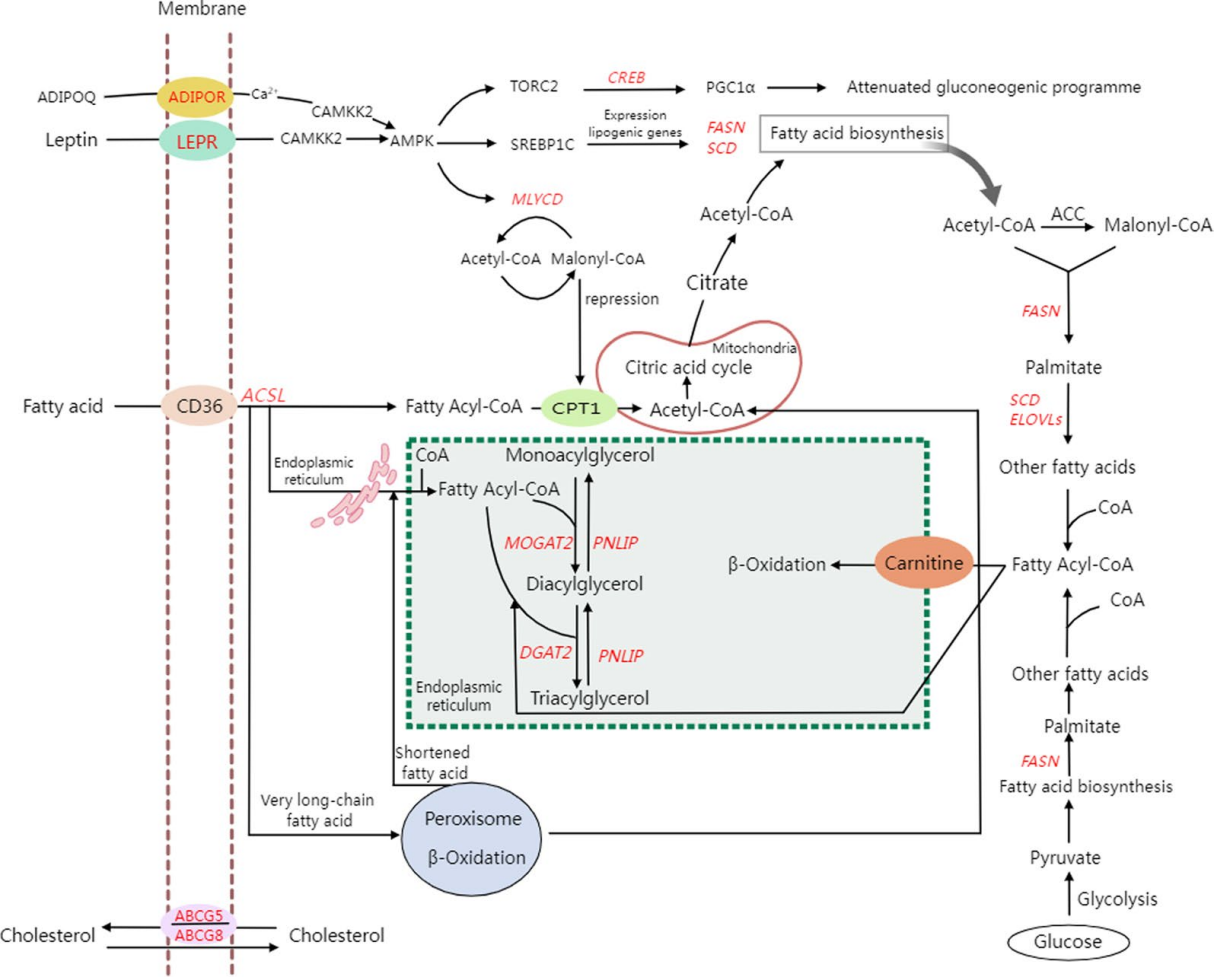
Candidate genes in significant QTL regions and their role in fatty acid metabolic pathways

GWAS of the FA traits identified associated genomic regions and candidate genes for FA metabolism, which are involved in multiple metabolic pathways (Fig. 2). For example, ADIPOR and LEPR, respectively, transport adiponectin and leptin to the cytoplasm to take part in the AMPK signaling pathway. AMPK signaling is a key pathway that regulates energy metabolism by reducing anabolism and enhancing catabolism to increase the intracellular ATP reserve, including the downregulation of synthetic fat genes, cholesterol, and FAS and the upregulation of glucose and fat transport, FAs oxidation, and oxidative metabolism [89]. Henriquez-Rodriguez et al. [90] reported that the SNP NM_001024587:g.1987C > T, which is located in exon 14 of *LEPR* was significantly associated with SFA, MUFA, and PUFA in Duroc pigs. Another SNP located in exon 15 of *LEPR* (rs709596309) was reported to affect the circulating amount of C18:1*n*-9 in fasting Duroc pigs, reflecting the metabolic status of an animal at a given time [91]. The latter SNP was also significantly associated with the contents of C18:1*n*-9, SFA, MUFA, and C18:1*n*-9/C18:0 in our study. In addition, these FA traits were also significantly associated with QTL region A14 in our study, which contains *ELOVL3* and *SCD*.

The candidate genes *FASN, SCD,* and *ELOVL*s are involved in FA biosynthesis. C16:0 is catalyzed by *FASN*, followed by the *ELOVL6* elongation to produce C18:0. Furthermore, *ELOVL3* is involved in elongation of SFAs and MUFAs, and *ELOVL5* is responsible for elongation of PUFAs [92]. Additionally, recent findings suggest that *DGAT2* is specifically involved in de novo adipogenesis by incorporating endogenous FA into TAG, specifically MUFA that is generated by the desaturation of C16:0 and C18:0 through the action of *SCD* [44]. This finding is consistent with our results, as the abundance of the desaturated products from C16:0 and C18:0 in backfat are significantly associated with the QTL on SSC14, where the *SCD* is located. The *MOGAT2* gene, annotated on QTL A9 (SSC9: 0–14.55 Mb), is also involved in adipogenesis and is a key sensor of energy metabolism in response to dietary fat. Under a high-fat diet, *MGAT2*-lacking mice showed better glucose tolerance and protected organisms from hepatic steatosis [93]. The *PNLIP* and its family members (*PNLIPRP1*, *PNLIPRP2*, *PNLIPRP3*) are involved in the hydrolysis of TAG, particularly targeting unsaturated substrates [69]. This function of the *PNLIP* gene is concordant with the identification of a QTL for the SFA content in the genomic region where *PNLIP* is located. The *ACSL5* gene was annotated only in the QTL for C18:0, its precursor, and its unsaturated products in muscle. It is localized in the outer membrane of endoplasmic reticulum and mitochondrial, where it catalyzes the formation of fatty acyl-CoAs from LCFA for beta-oxidation [68]. Although deducing FA metabolic pathways contributes to understanding the functional role of candidate genes identified in GWAS, experimental works are needed for the functional validation of genetic variants and genes.

### Tissues cross talk in the control of fatty acid metabolism

The C20:4*n*-6/C20:3*n*-6 ratios in liver, muscle, and backfat were associated with the 7.88–13.74 Mb genomic region on SSC2 in our study. The same genomic region was associated with the backfat content of DGLA and C22:4*n*-6 and the muscle C18:3*n*-6/C18:2*n*-6 ratio. In this region contains genes that encode the rate-limiting enzymes FADS2 and FADS1, which are involved in the desaturation of C18:2*n*-6 to form other N6 PUFAs. In addition, in backfat, the percentage of C18:2*n*-6 in liver was positively correlated (r = 0.61) with AA content in our study. Dietary fat is digested by lipase or bile salts, releasing the C18:2*n*-6 FA, which is absorbed by the small intestine. The absorbed C18:2*n*-6 is then re-esterified into TAG or into phospholipids or cholesteryl esters, which are assembled to chylomicrons and enter the circulation for delivery to other tissues [94]. Dietary C18:2*n*-6 is transformed by FADSs and ELOVL5 enzymes to AA and DGLA in the endoplasmic reticulum of liver cells. Adipose tissue and muscle can obtain pre-formed PUFA from hepatic synthesis or from the diet by blood circulation [95]. Furthermore, the composition of C18:2*n*-6 in adipose tissue greatly influences adipocyte differentiation and thermogenesis by regulating the conversion of AA [96]. And with fat deposition, the proportion of AA in adipose tissue and muscle decreases. In addition, we observed that backfat had the lowest C20:4*n*-6/C20:3*n*-6 ratio and the highest C20:2*n*-6 concentration in comparison with the other three tissues investigated. This result can be explained by a major conversion of C18:2*n*-6 to C20:2*n*-6 by ELOVL5 elongation, followed by two consecutive desaturations with FADS2 and FADS1 to generate AA. Therefore, the observed FA profile in backfat may represent a combined outcome of FA biosynthesis and oxidation in liver, adipose tissue, and muscle. Furthermore, blood showed a high C20:4*n*-6/C20:3*n*-6 ratio, but no QTL was detected for this trait. Hence, this result can be explained by C20:3*n*-6 to C20:4*n*-6 desaturation activity in liver. In this sense, the high C20:4n-6/C20:3n-6 ratio we observed in the liver suggests desaturase activity in this tissue is producing AA from DGLA FAs. When the level of the essential C18:2*n*-6 is high in the organism, it promotes its downward conversion to DGLA, which can be desaturated to AA. When a certain level of AA is produced, negative feedback regulation may occur [97].

The percentages of C18:0, C18:1*n*-7 and of SFA, as well as the C16:1*n*-7/C16:0 and C18:1*n*-9/C18:0 ratios in both muscle and backfat were strongly associated with the 100.91–128.14 Mb genomic region on SSC14, although several other genomic intervals were also associated with these FAs. Moreover, the percentages of C16:0 (r = 0.81), C16:1*n*-7 (r = 0.65), and C18:1n-9 (r = 0.91) were positively correlated between muscle and adipose tissue. The relative composition of these FAs is affected by the desaturation activity of the *SCD* gene, which is located within the SSC14 region. SCD is localized in the endoplasmic reticulum and plays a pivotal role in partitioning endogenous and dietary FAs into metabolically active or inactive pools [98]. Inhibition of SCD activity leads to elevated FA oxidation and decreased lipid synthesis and storage, attributed to downregulation of lipogenic genes [99]. As a key enzyme in MUFA generation, SCD catalyzes the rate-limiting step in de novo synthesis of C18:1*n*-9 and C16:1*n*-7 from their precursors, the C18:0 and C16:0 FAs, respectively, through Δ9-desaturation. Additionally, C18:1*n*-7, as a natural trans FA, can be metabolized by SCD into cis-9, trans-11 conjugated linoleic acid, and elaidic acid, which has been demonstrated to disrupt C18:1*n*-9 synthesis [100]. We also found that the C18:1*n*-9/C18:0 ratio was higher in both muscle and backfat than in blood and liver, suggesting higher SCD activity in these tissues. Silva-Santi et al. [101] found that C16:1*n*-7 derived from adipose tissue acts as a lipokine, downregulating hepatic lipogenesis and increasing insulin sensitivity in peripheral tissues. In addition, the 146.05–148.36 Mb genomic region on SSC6, which contains the *LEPR* gene, was associated with C18:1*n*-9 content and the C18:1*n*-9/C18:0 ratio in backfat. Leptin is the ligand of LEPR and it is an adipokine with a major role in regulation of energy homeostasis and other physiologic functions. Mauvoisin et al. [102] demonstrated that, in HepG2 cells, leptin inhibits the expression of the *SCD* gene by inhibiting the ERK1/2 MAPK pathway, which targets the transcription factor Sp1 on the *SCD* gene promoter.

The C18:0/C16:0 elongation ratio in blood was associated with the 118.92–123.95 Mb genomic region on SSC4, which contains the *ABCD3* gene, and with the 65.31–73.74 Mb region on SSC14, where the *ARID5B* gene is located. However, in backfat the C18:0/C16:0 ratio was associated with two different genomic regions on SSC14, at 103.81–115.64 Mb and 129.01–139.72 Mb, in which the *ELOVL3* and *ACADSB* genes are located, respectively. It is widely recognized that *ELOVL6* gene is involved in elongation of C16:0 to C18:0, but we did not detect any association of SNPs in the *ELOVL6* genomic region with the C18:0/C16:0 ratio. Notably, the concentration of C16:0 (r = 0.85) and C18:0 (r = 0.68) in blood were both positively correlated with the C16:0 content in liver. Compared to other tissues, the C16:0/C14:0 and C18:0/C16:0 ratios were higher in liver, suggesting than liver is more active in the elongation of these FAs. Regulation of de novo FAS is highly synchronized between the liver and adipose tissue, in response to feeding and fasting under physiological conditions [103]. However, this coordination is disrupted by obesity, which increases de novo FAS activity and FA esterification in liver, resulting in higher triglyceride levels in circulation [103]. Meanwhile, the ability of de novo FAS in adipose tissue is reduced. Moreover, the disruption of coordination between the liver and adipose tissue progressively intensifies during the fattening of pigs. Additionally, the genomic region in which the *ELOVL3* gene is located (SSC14: 103.81–115.64 Mb) was associated with the contents of C18:0 and C18:1*n*-7 both in backfat and muscle. The formation of C18:0 has been discussed previously, while the production of C18:1*n*-7 is catalyzed by the *ELOVL6* elongase from C16:1*n*-7. The *ELOVL6* gene is involved in the elongation of MUFA, together with *ELOVL3*. Although C16:1*n*-7 and C18:1*n*-7 levels were higher in backfat and muscle compared to the liver and blood, in our study, the elongase activity from C16:1*n*-7 to C18:1*n*-7 was lower in these tissues than in the liver and blood. Activity of *ELOVL* gene family is transcriptionally regulated, and differential transcriptional regulation suggests that genes from this family may be involved in different synthetic pathways by affecting different cellular FAs pools, in response to various stimuli [104]. The *ELOVL6* gene is the target for SREBP-1c and LXRα, which regulate FA synthesis in a lipogenic manner, while expression of the *ELOVL3* gene is inhibited by LXR but can be induced by factors that stimulate FA oxidation [104]. In activated brown adipose tissue of mice, induction of the *ELOVL3* gene promotes FA oxidation during cold exposure. The FAs elongated by activity of the *ELOVL3* gene are used to replenish intracellular pools of FAs when the FA turnover rate is high [104, 105].

### Most promising candidate genes involved in energy metabolism and immunity

As discussed above, the AMPK system acts as a sensor of cellular energy status and is important for homeostasis of the organism by stimulating catabolic pathways to produce ATP and by inhibiting biological pathways that consume ATP [106]. Immunological and environmental stimuli can promote activation of AMPK in T cells, and further impact inflammation that is mediated by T cells [106]. Activated T cells promote de novo synthesis of FAs, which involve the acetyl-CoA citrate lyase (*ACLY*), *ACC1,* and *FASN* genes [107]. However, less is known about the role of other FAS genes in controlling the fate of T cells, such as *SCD* and *ELOVLs*.

GATA3 is a transcription factor that is involved in innate and adaptive immunity as an immunomodulator that regulates T cell differentiation, development, proliferation, and maintenance by controlling target gene expression to regulate cellular function [108]. For instance, El-Arabey et al. [109] reviewed that the expression of *GATA3* in adipose tissue promotes infiltration of immune cells into muscle by downregulating IL-10 and into liver by releasing IL-6 and leptin. In our study, the *GATA3* gene was located in QTLs for the C20:4*n*-6/C20:3*n*-6 ratio in liver and backfat.

Candidate genes and pathways identified by GWAS for blood FA traits are limited. Among them, *ABCD3* has been proven to participate in immune response regulation [110]. Similarly, the *ABCD2* gene, found in QTL identified in the GWAS of FA in backfat, is also involved in immune response. The ABCD2 and ABCD3 proteins are localized on peroxisomes and are associated with proliferation and activation of immune cells. In recent years, peroxisomes have been proposed to be involved in inflammation and antimicrobial responses due to its properties of scavenging reactive anionic species, cellular redox status, degradation of inflammation modulators, such as AA-derived prostaglandins or leukotrienes, and its involvement in PUFA metabolism [111]. In addition, peroxisomes not only produce metabolites to control immune pathways but also recruit and activate signaling proteins in response to a stimulus [111]. Therefore, other proteins involved in oxidation warrant a more thorough investigation to understand their roles in immune and metabolic processes, such as ACAA1, ACSL5, and MLYCD.

**Fig. 3:**
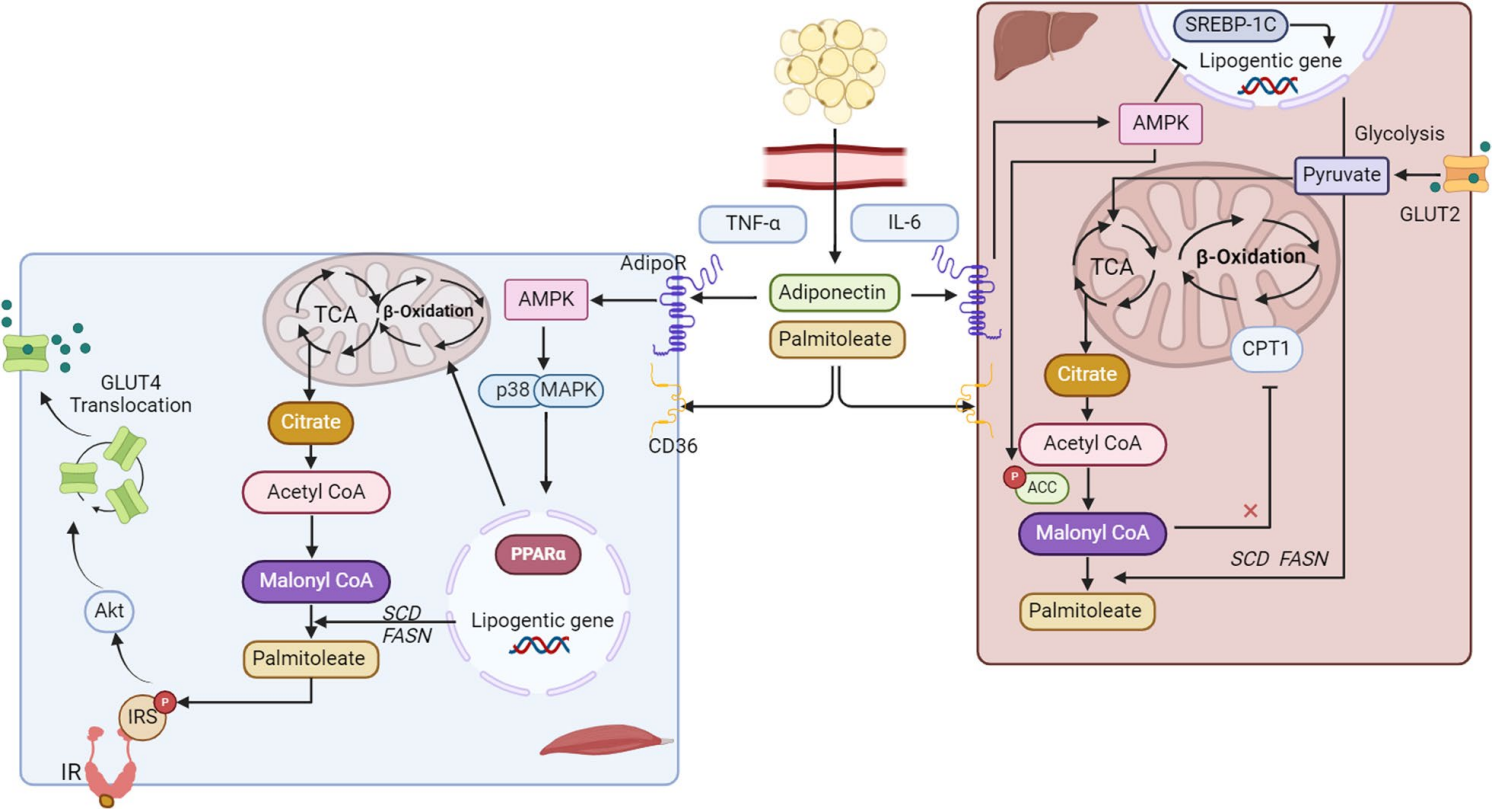
Inter-organ crosstalk regulated by adipokines. Adipokines are released into circulation by adipose tissue and target muscle and liver. In muscle, adiponectin activates the p38 mitogen-activated protein kinase (MAPK) and peroxisome proliferator-activated receptor alpha (PPARα), stimulating fatty acid (FA) oxidation. High expression of the *FASN* and *SCD* genes promotes the production of palmitoleate, which increases the phosphorylation of insulin receptor substrate (IRS) and facilitates the translocation of the Glucose transporter type 4 (GLUT4) via Akt (protein kinase B) to assist glucose uptake. In the liver, adiponectin activates AMP protein kinase (AMPK) and decreases SREBP1c expression to inhibit hepatic lipogenesis. In addition, activated AMPK phosphorylates acetyl CoA carboxylase-1 (ACC1), which inhibits and decreases the production of malonyl-CoA and promotes FA transport into mitochondria for beta-oxidation

As an endocrine tissue, adipose tissue secretes signaling molecules known as adipokines, such as leptin, adiponectin, FA esters of hydroxy FAs, tumor necrosis factor alpha (TNF-α), and interleukin-6 (IL-6) (Fig. 3) [112]. Palmitoleate is an adipokine that is involved in inter-organ signaling in metabolism, in activating lipogenesis, and in regulating FA accumulation [113]. Upregulation of *FASN* and *SCD* genes in muscle increases de novo synthesis of palmitoleate, which can increase insulin sensitivity and glucose uptake by promoting phosphorylation of insulin receptor substrate and Akt [113]. Moreover, a study in C2C12 myotubes reported that palmitoleate alleviates inflammation by delaying proinflammatory marker cyclooxygenase-2 activation [114]. In addition, SFAs are known to have proinflammatory effects, trigging NFκB and activating atherosclerosis. SFAs promote expression of cell adhesion molecules to increase the adherence and transmigration of monocytes, while also elevating the levels of inflammatory cytokines (TNF-α, IL-6, and IL-8), thereby further inducing endothelial cell apoptosis, which is the initial step of atherogenesis [115]. Within the interaction between adipose tissue and muscle, adiponectin stimulates FA oxidation via activation of p38 MAPK and peroxisome PPARα, by decreasing intramuscular lipid accumulation, and by preserving insulin sensitivity [116]. Whereas, in liver, adiponectin decreases hepatic lipogenesis and enhances beta-oxidation via the activation of AMPK and PPARα [117]. Activated AMPK phosphorylates ACC1, which is a rate-limiting enzyme of de novo lipogenesis, inhibiting lipogenesis. Reduced ACC1 activity decreases malonyl-CoA production, thereby inhibiting the activity of carnitine palmitoyl transferase-1 and facilitating FA transport into mitochondria for beta-oxidation [112].

Energy metabolism is a complex process, which is dynamically remodeled according to the metabolic and health state. Adipose tissue act as storage depot across numerous organs and plays regulatory roles in survival, reproduction, and adaptation. The Duroc pigs used in our study contain a higher fat content in comparison to other breeds, which may result in the different characteristics of fat depots, such as variations in adipocyte size and number [118]. These differences subsequently influence the secretion of adipokines. The alternations in circulating levels of adipokines may lead to different response in energy metabolism, particularly under conditions of metabolic stress and inflammation. Because our study was conducted on healthy pigs, validating our findings across different environments, stressors, and breeds will be challenging.

### Links between candidate genes and meat quality

In recent years, consumers have become more concerned about the quality and healthfulness of meat. Meat quality is affected by multiple factors, notably by fat content and its FA composition, which impact flavor, color, firmness, and softness of meat. From a nutritional perspective, excessive consumption of SFA increase the risk of cardiovascular diseases and type 2 diabetes, whereas replacing them with MUFA or PUFA mitigates these risks. Our GWAS identified several candidate genes that directly or indirectly regulate intramuscular fat (IMF) content, including the *FABP1*, *SCD*, *FASN*, *FADSs, ELOVL3,* and *LEPR* genes. IMF, commonly known as marbling, serves as a pivotal indicator for meat quality.

FABPs facilitate the intracellular transport of FAs, significantly influencing the uptake, transport, and esterification of FAs. Of these, the *FABP1* gene, identified for its beneficial effect on meat quality in Berkshire pigs through selection [119], influences the uptake, transport, and esterification of FAs. FABP1 exhibits a preference for binding N3 PUFAs and plays a key role in mediating the activation by N3 PUFA and PPARα target genes involved in beta-oxidation [34, 119]. In our study, a QTL that contains the *FABP1* gene was identified for the C16:0/C14:0 ratio in backfat, but not for N3 PUFAs. In fact, as shown in Additional file 4: Figure S1(G), the ratio C16:0/C14:0 was also strongly associated with the QTL A9, which includes the *DGAT2* gene. Therefore, the *FABP1* gene may be involved in TAG formation, together with *DGAT2* gene, and affect pork quality.

*SCD* is another regulatory gene, which expression is positively correlated with IMF content (r = 0.48), that participates in the PPAR signaling pathway. SCD is a rate-limiting enzyme for biosynthesis of MUFA from SFA and regulates lipogenesis [120, 121]. An increase in IMF is associated with a higher level of MUFA, mainly oleic acid, which is an important marker for meat quality. Our study identified the *SCD* gene to be located in a QTL for the ratios of C16:1*n*-7/C16:0 and C18:1*n*-9/C18:0 in both muscle and backfat. Similarly, *FASN* is a gene involved in the fat synthesis and deposition and a polymorphism (rs326206604) in this gene was significantly associated with backfat thickness in Polish Large White pigs [122]. This SNP was significantly associated with C14:0 content (*p*-value = 7.21E−10) and with the C16:0/C14:0 ratio (*p*-value = 4.13E−07) in backfat in the current study. However, excessive SFA accumulation leads to lipotoxicity [123]. To prevent adverse metabolic profiles, de novo synthesized SFA, particularly the C16:0, will activate mitochondrial depolarization, cell apoptosis, and suppress autophagy and lipid droplet formation [123]. Additionally, differentiated adipocytes protect themselves from cytotoxic effects by promoting the synthesis of C18:1*n*-9, involving elongation and desaturation mediated by the *ELOVL* and *SCD* [103].

The *FADS2* gene is involved in desaturation of C18:2*n*-6 to AA. The polymorphism rs321384923 in the promoter of *FADS2* modifies desaturation efficiency, thereby altering the N3 PUFA profile in a Duroc pig line [124]. This genetic variant has been proposed as a candidate marker to increase IMF without altering lean weight and was identified to be significantly associated with the ratio of C20:4*n*-6/C20:3*n*-6 (*p*-value = 1.12E−06) in muscle in our study.

The level of expression of *ELOVL3* has been reported to be highly correlated with IMF in chicken pectoralis, as it increases the proportion of long-chain unsaturated glycerophospholipid molecules [125]. In our work, the 103.81–115.64 Mb genomic region on SSC14, which contains the *ELOVL3* gene, was associated with the contents of C18:0, C18:1*n*-7, and SFA in both backfat and muscle.

Similarly, pigs with higher IMF levels were found to exhibit higher levels of the *LEPR* gene expression in muscle, while having a higher content of SFA and a lower level of PUFA [126, 127]. This FA composition is associated with lower moisture and flavor scores since the changes in the FA profile can lead to differences in melting points, and further change the meat firmness [126, 127]. Our study observed similar results, with the contents of C18:1*n*-9 and SFA in backfat being associated with the genomic region in which where *LEPR* gene is located. In addition, leptin, the ligand of LEPR, induces intracellular signaling in preadipocytes and adipocytes to promote adipogenesis and regulates energy homeostasis through tissue crosstalk by stimulating FA oxidation and glucose uptake [113].

## Conclusions

Our results described the FA composition in four relevant tissues for FA metabolism and energy homeostasis. A total of 25 regions on 15 chromosomes of pig genome that were associated with FA composition traits across blood, liver, adipose tissue, and muscle were identified. Four of these regions were identified to be associated with FA related traits in more than one tissue. In addition, 49 candidate genes were annotated in the identified associated regions. These not only include genes that are related to lipid metabolism, but also genes involved in energy production, maintaining membrane structure, participating in signaling pathways, and regulating inflammation by promoting lipogenesis. However, the functional validation of regulatory variants of these genes will be necessary in future works that consider the genetic correlations between different FA and the expression of these genes. Overall, our findings were consistent with previous studies and identified new QTL regions that were closely related to energy metabolism, providing a reference for future genetic breeding selection to improve health and production-quality traits in pigs.

## Supplementary Information

Additional file 1: Table S1. Descriptive statistics summary of relative abundance of 31 individual FA traits and 25 FA indexes or ratios of liver, muscle, backfat, and plasma from Duroc pigs

Additional file 2: Table S2. The detailed top SNP list and annotated genes of genomic regions associated with fatty acid composition traits across four tissues

Additional file 3: Table S3. The summary of the most significant associated SNPs and their predicted consequences

Additional file 4: Figure S1. Manhattan plots and quantile–quantile plots representing the p-values profiles corresponding to the association analysis between fatty acid traits and SNPs,for plasma C16:0for plasma C18:0/C16:0for plasma N6for liver C20:4*n*-6/C20:3*n*-6for liver C20:1n-9/C18:1n-9for adipose C20:4*n*-6/C20:3*n*-6for adipose C16:0/C14:0for adipose C20:0/C18:0for adipose C18:1*n*-7for adipose C18:1*n*-9for adipose C14:0for adipose C22:4*n*-6for adipose C18:1*n*-9/C18:0for adipose C16:1*n*-7/C16:0for muscle C20:4*n*-6/C20:3*n*-6for muscle C16:0/C14:0for muscle SFAfor muscle C18:0 andfor muscle C18:1*n*-7. Red line indicates those SNPs that are below the genome-wide significance threshold.

## Data Availability

The data of the current study are available within the article and its supplementary materials. Additional data are part of a reference population used for genomic selection. Therefore, due to its commercial value, restrictions apply to the availability of these data. The corresponding author can be contacted for reasonable requests.
