# Supplementary figures and images for: Identification of genomic regions associated with fatty acid metabolism across blood, liver, backfat and muscle in pigs
Source: Genet Sel Evol. 2024 Sep 26;56:66. doi: 10.1186/s12711-024-00933-3 (PMC11426007; doi:10.1186/s12711-024-00933-3)

(A)


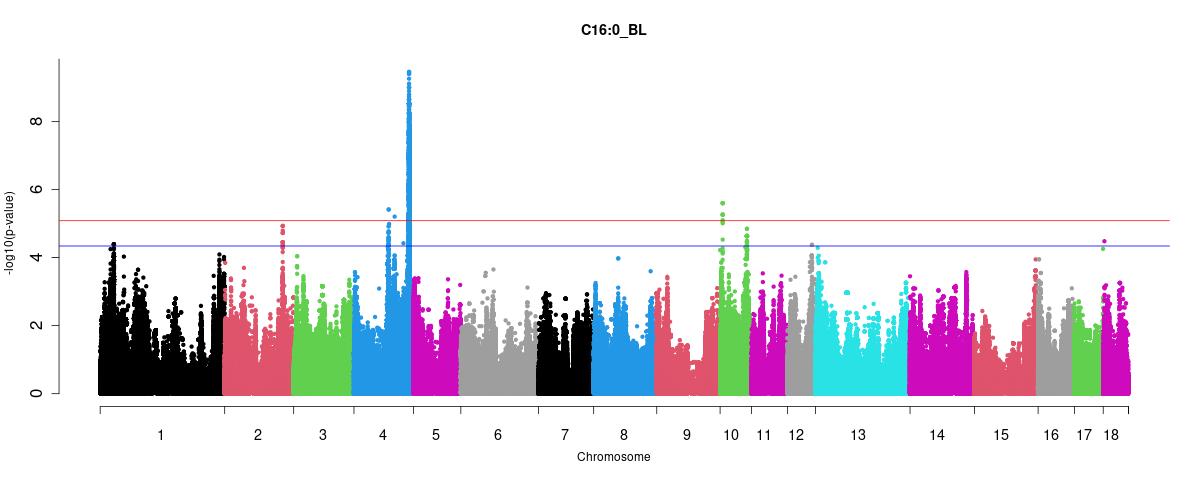

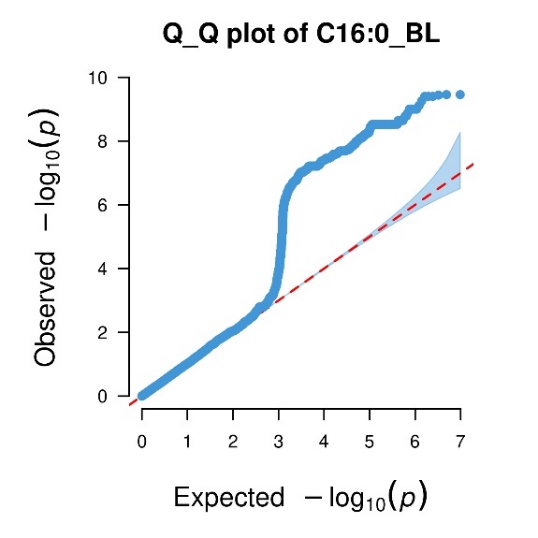


(B)


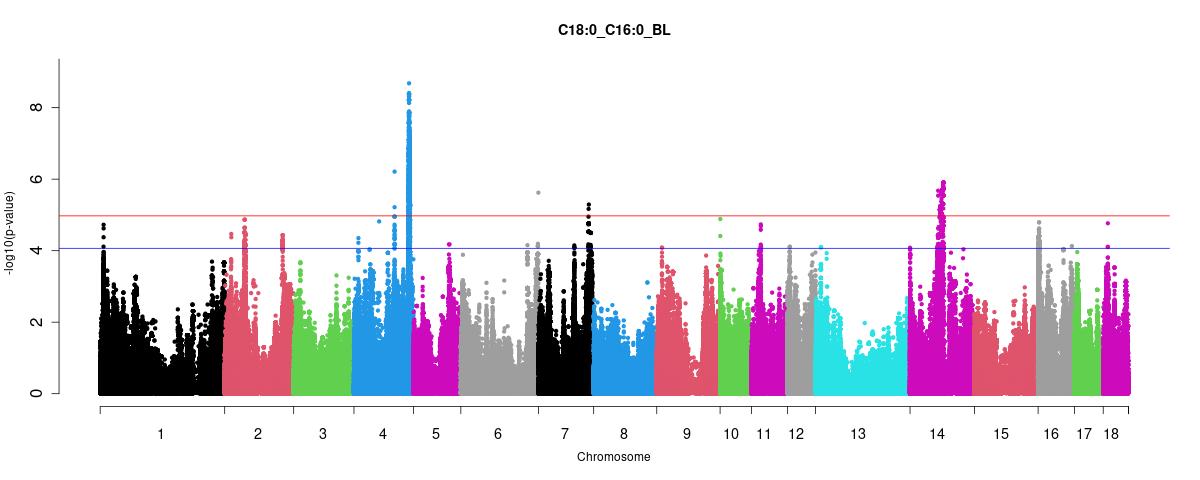

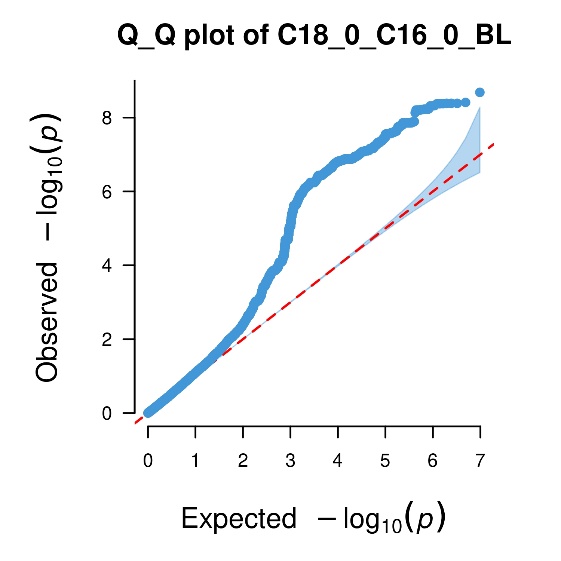


(C)


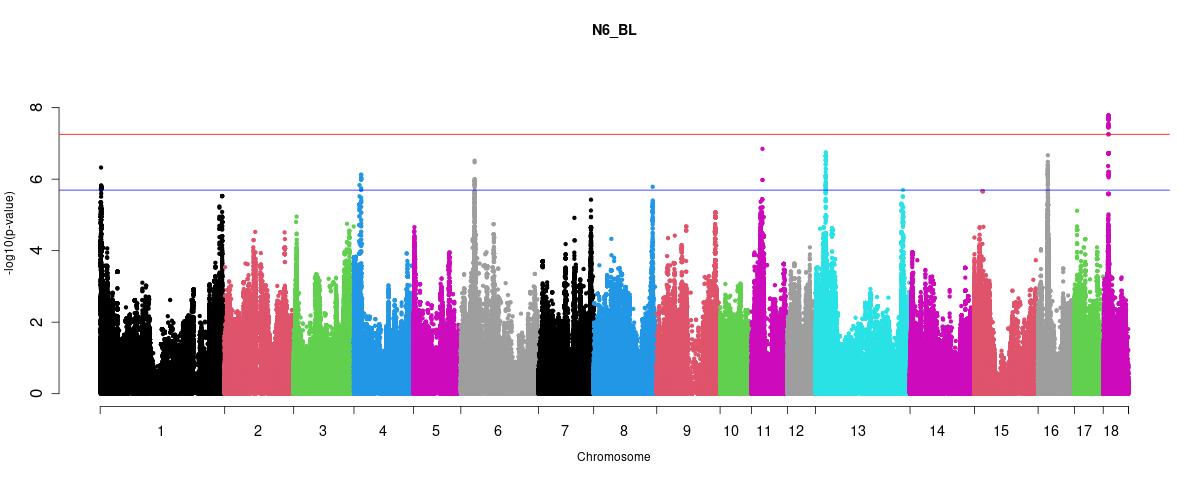

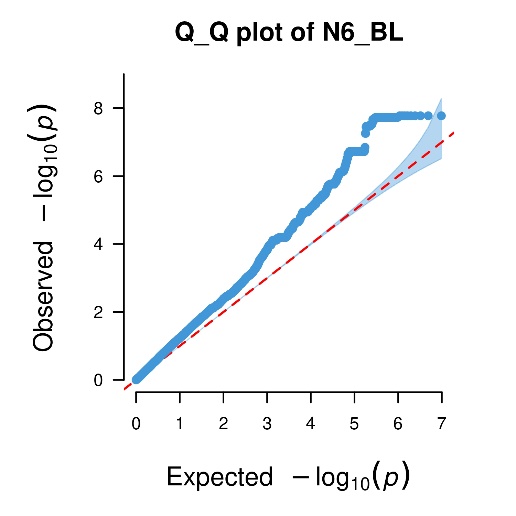


(D)


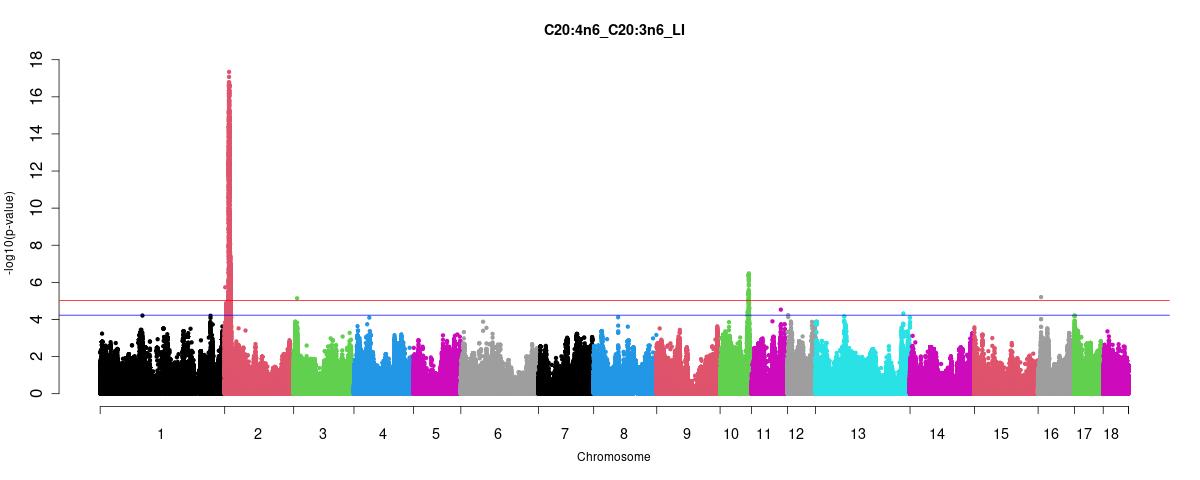

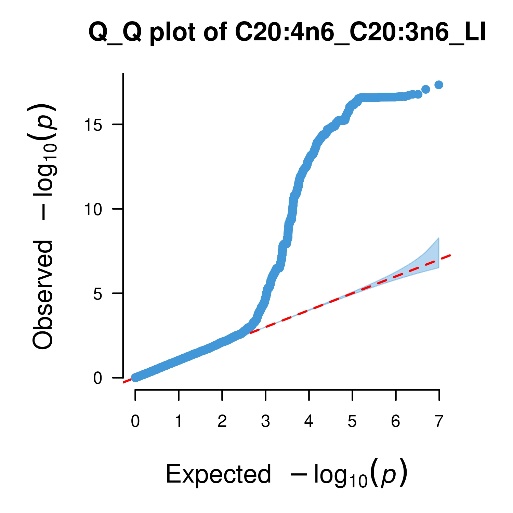


(E)


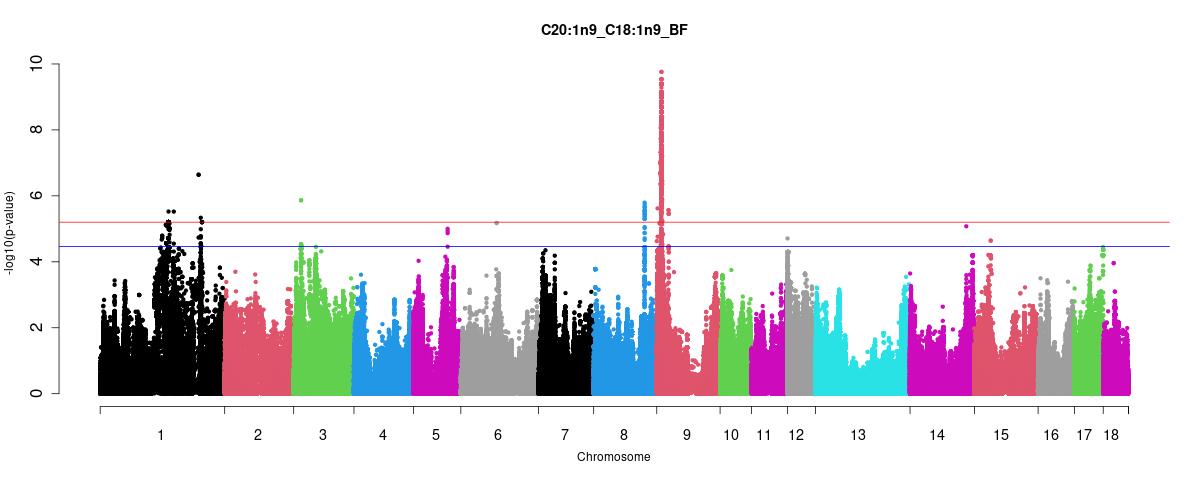

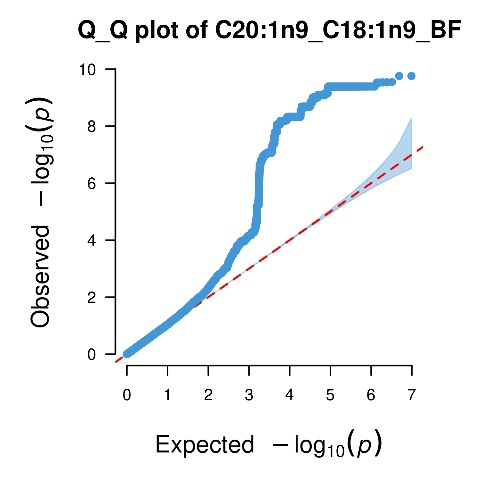


(F)


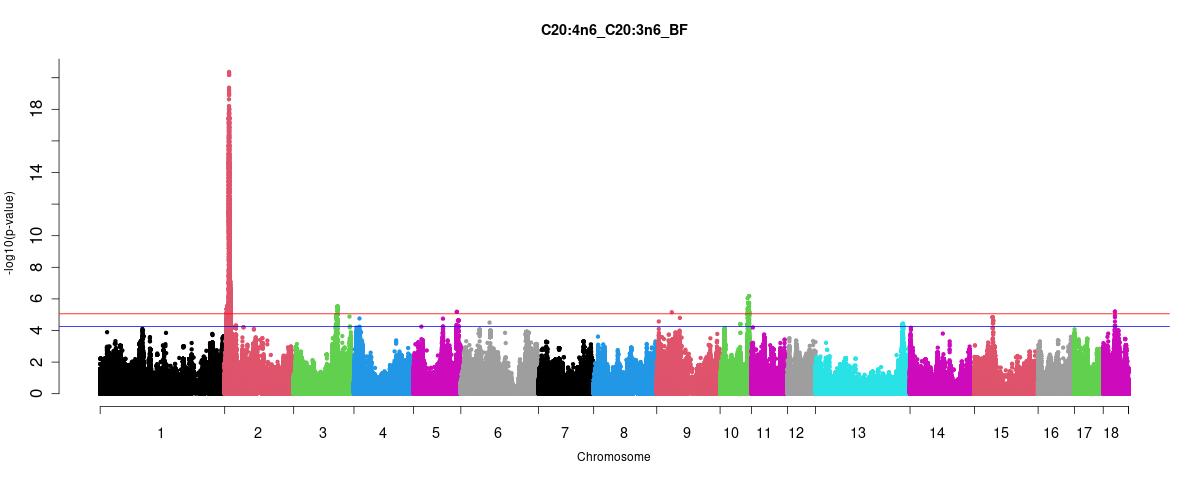

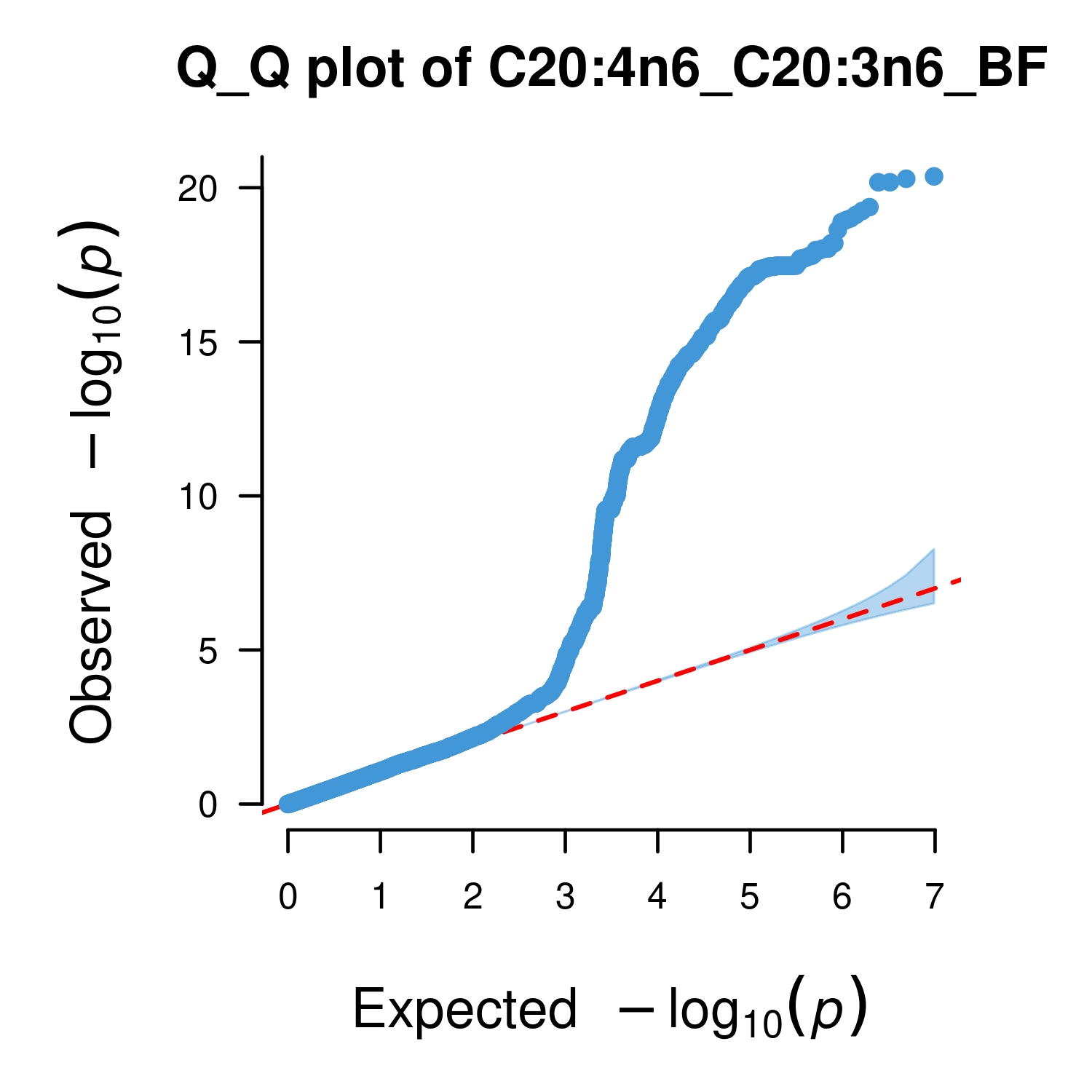


(G)


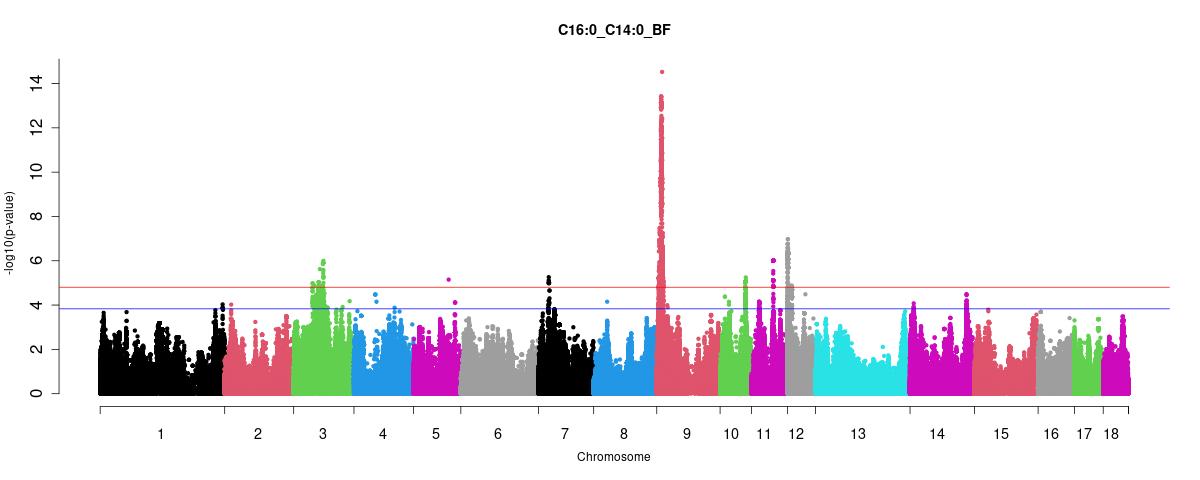

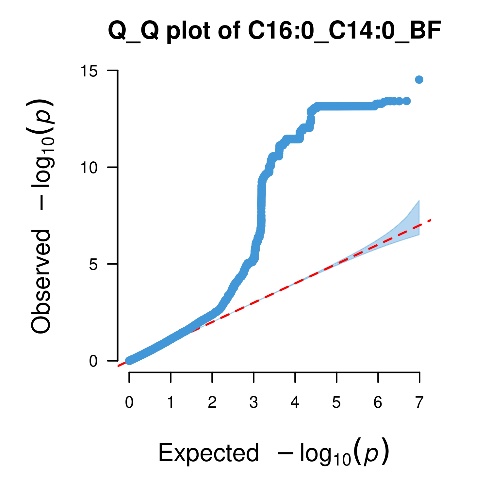


(H)


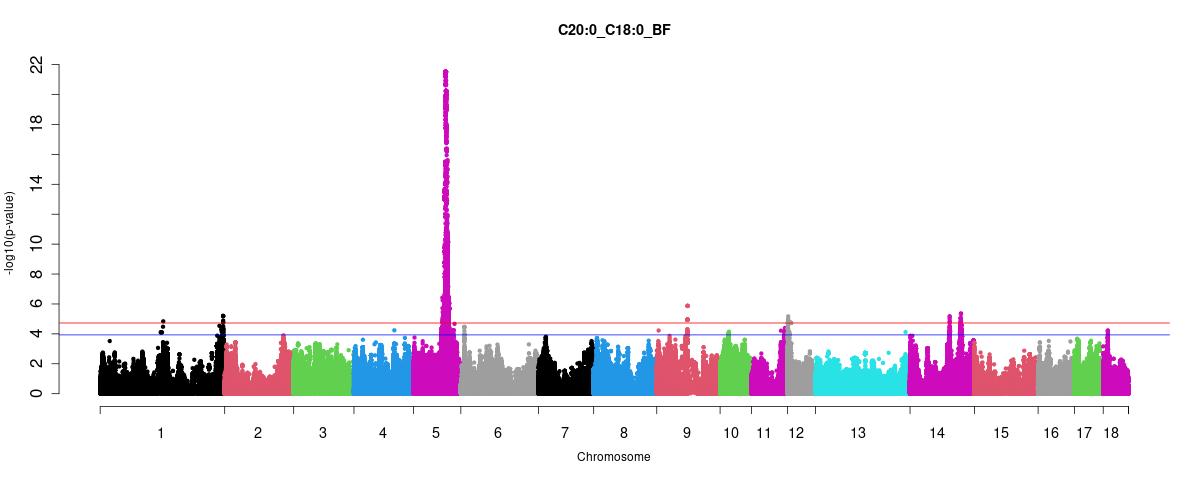

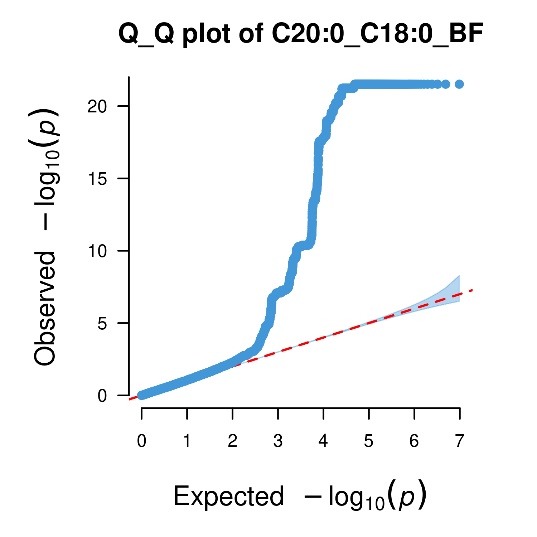


(I)


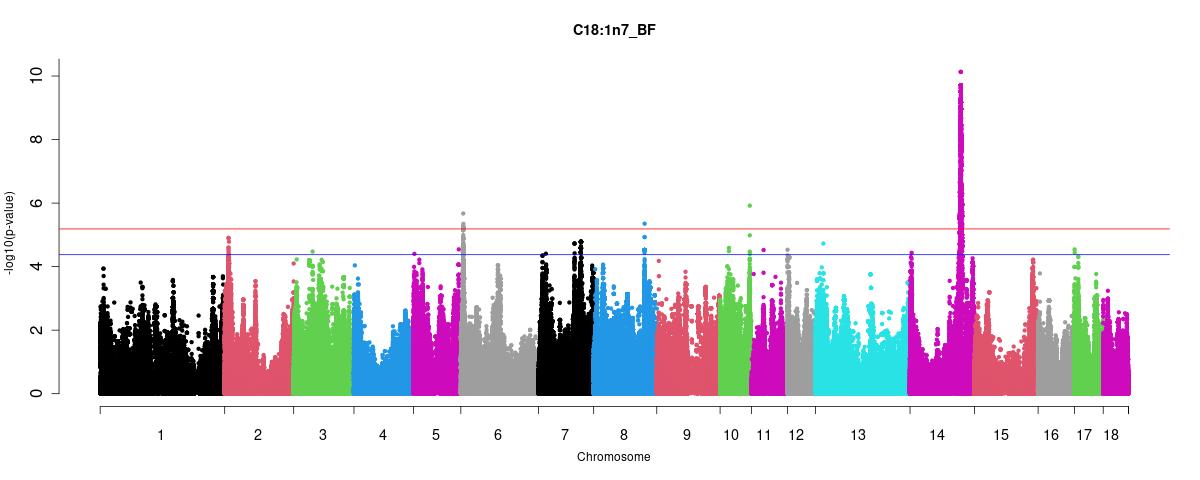

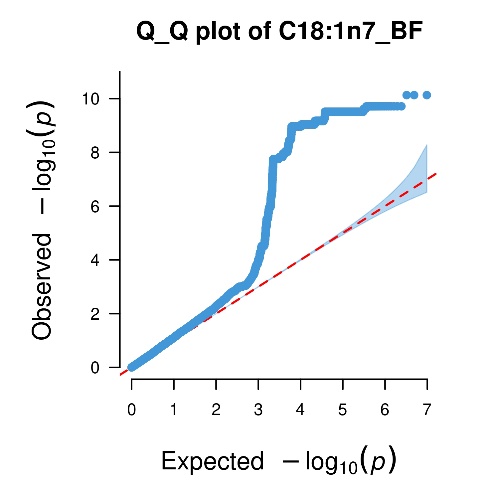


(J)


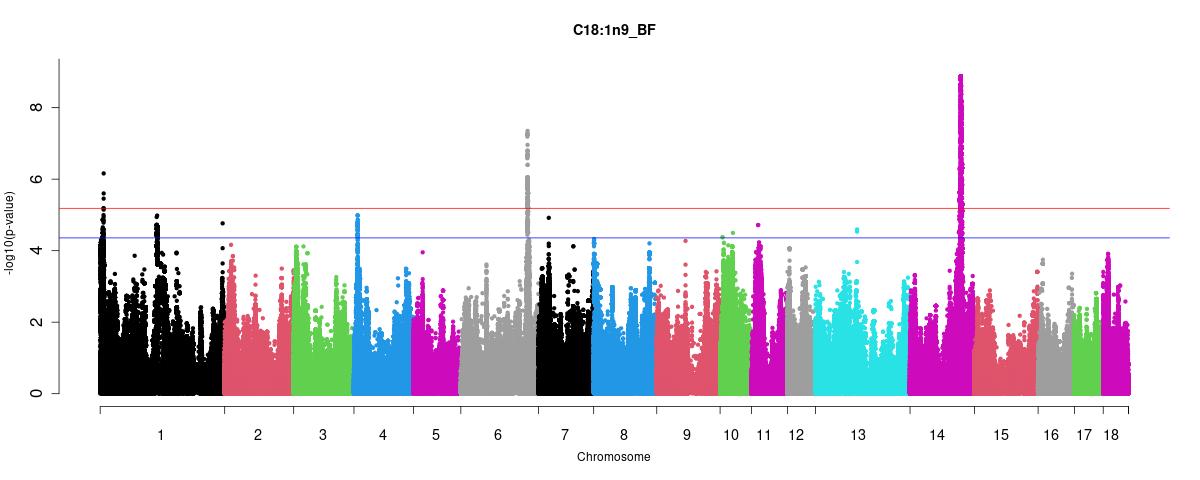

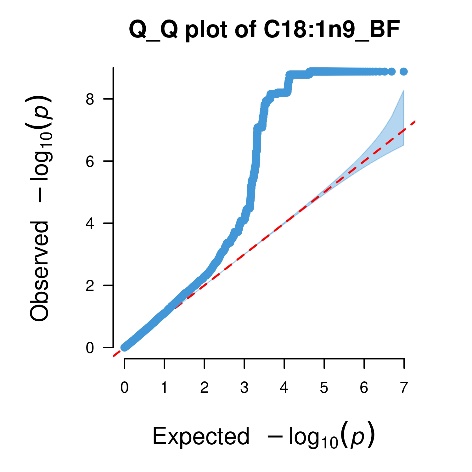


(K)


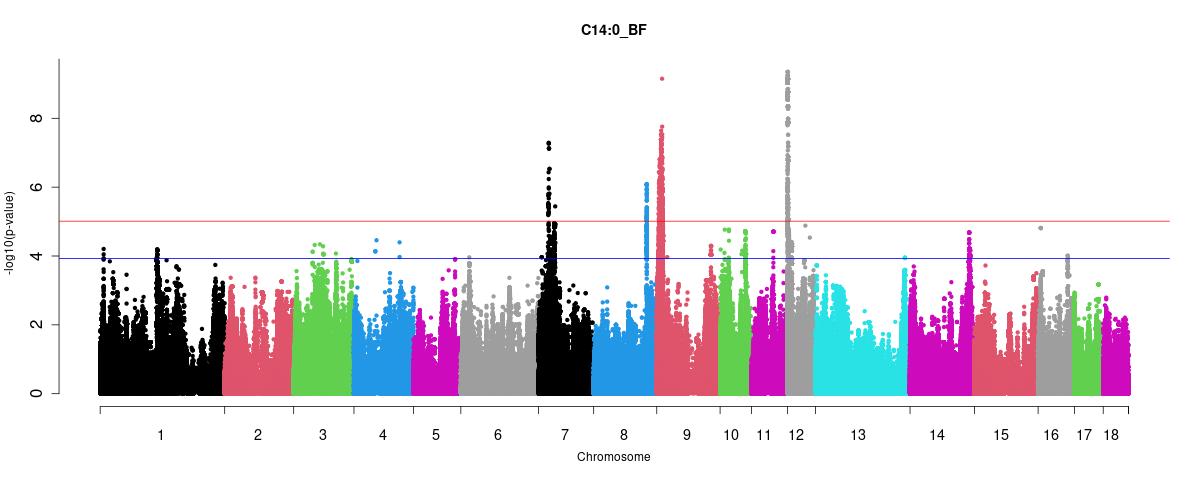

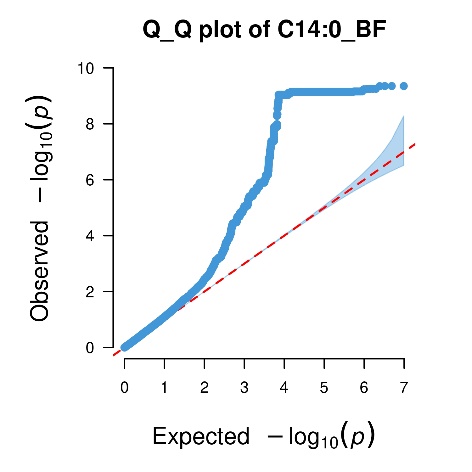


(L)


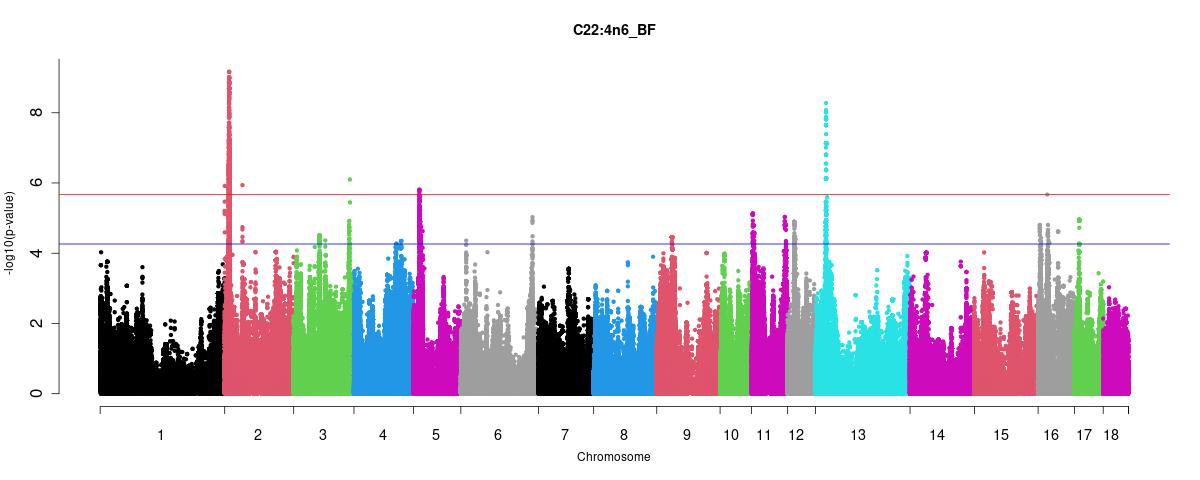

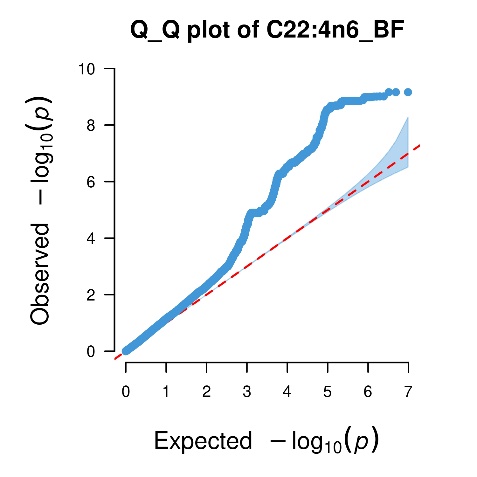


(M)


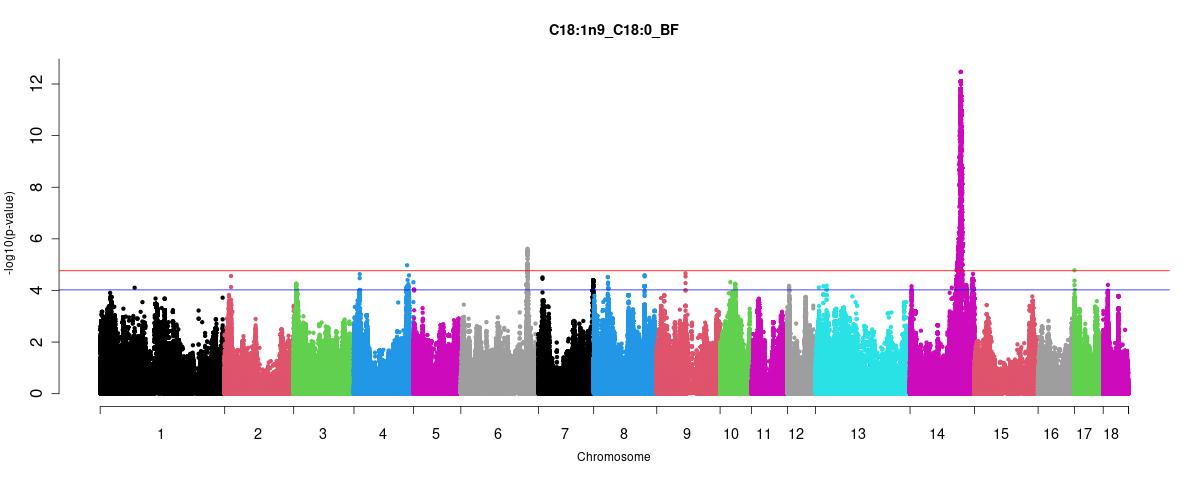

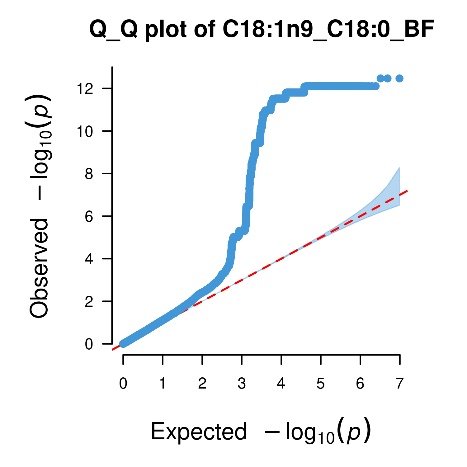


(N)


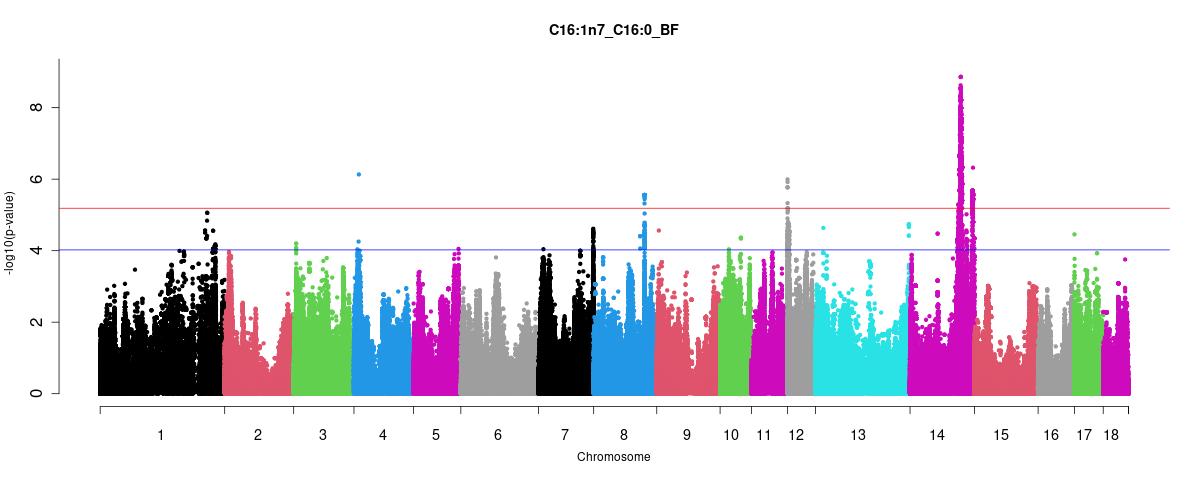

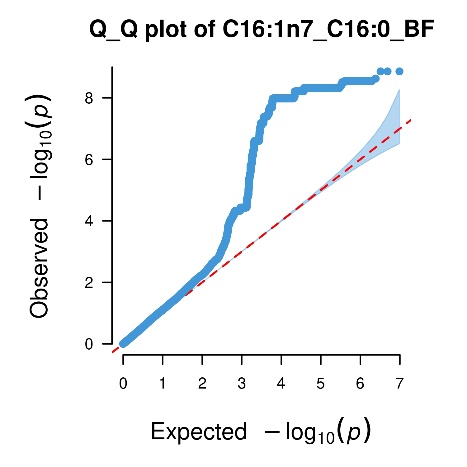


(O)


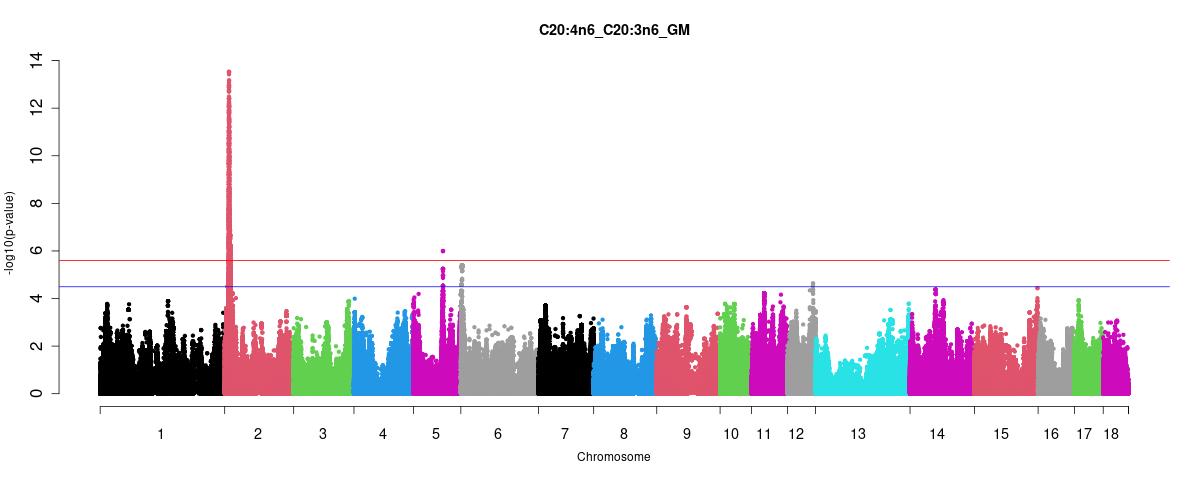

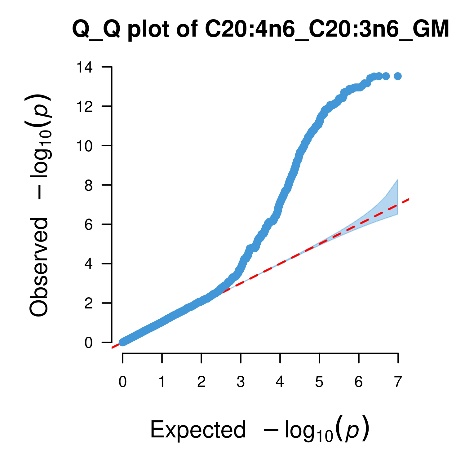


(P)


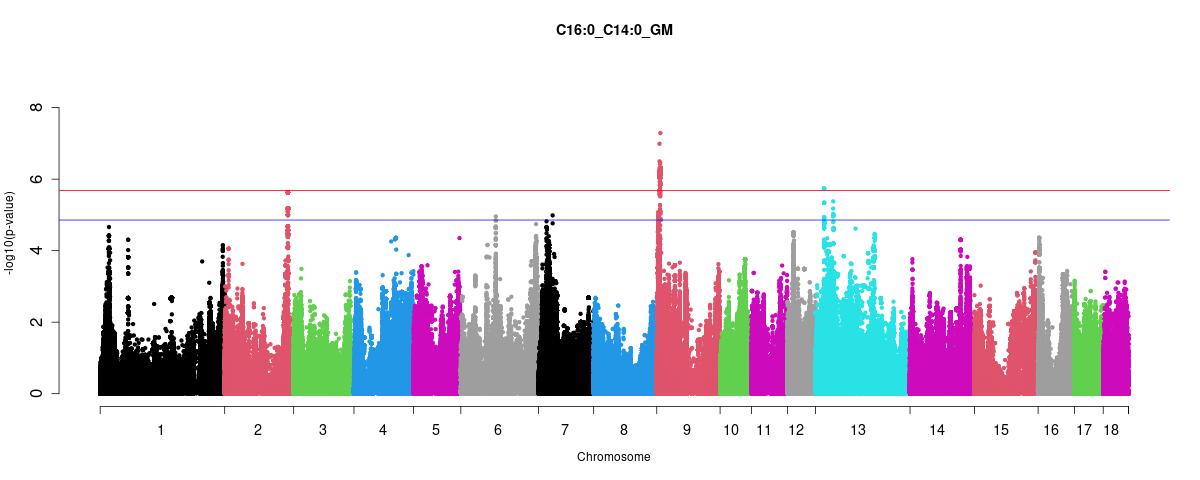

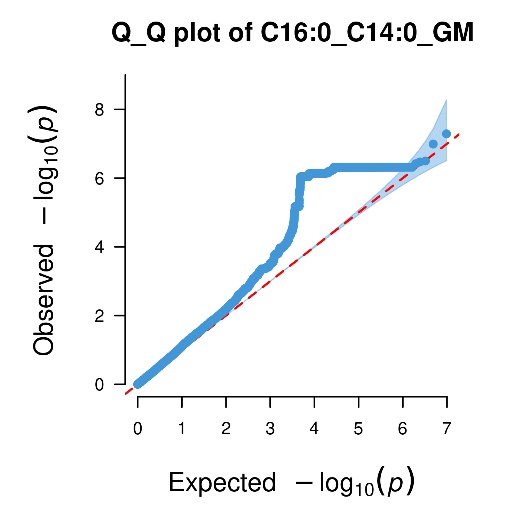


(Q)


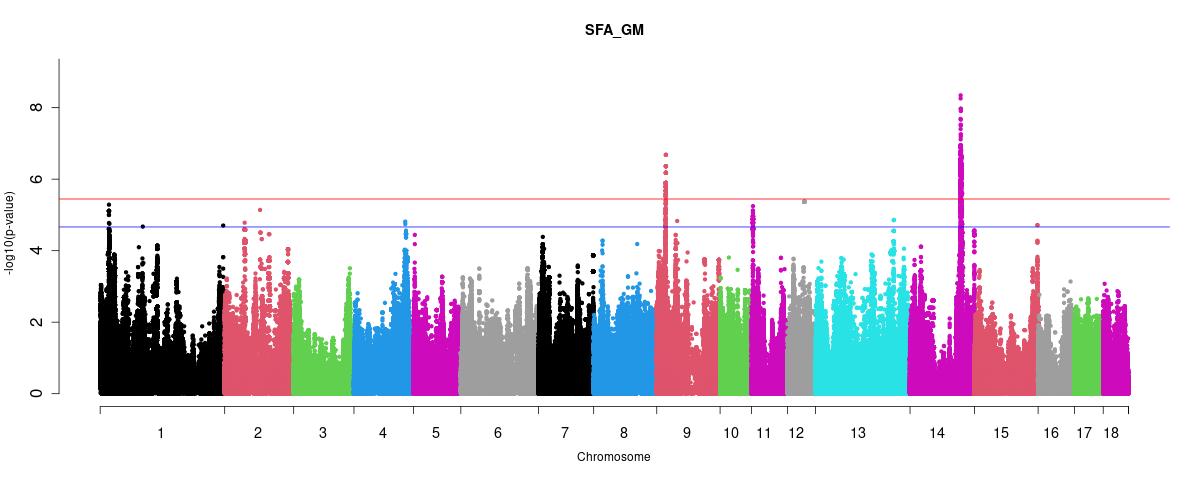

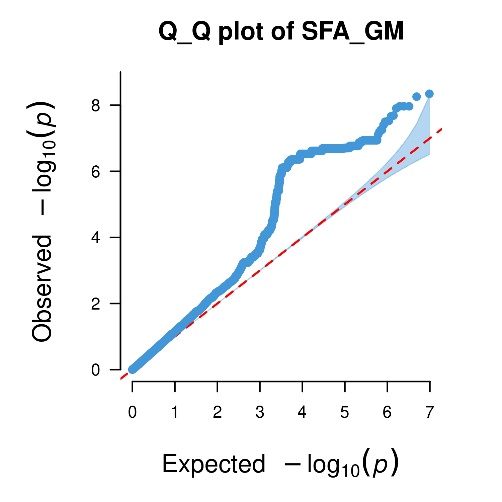


(R)


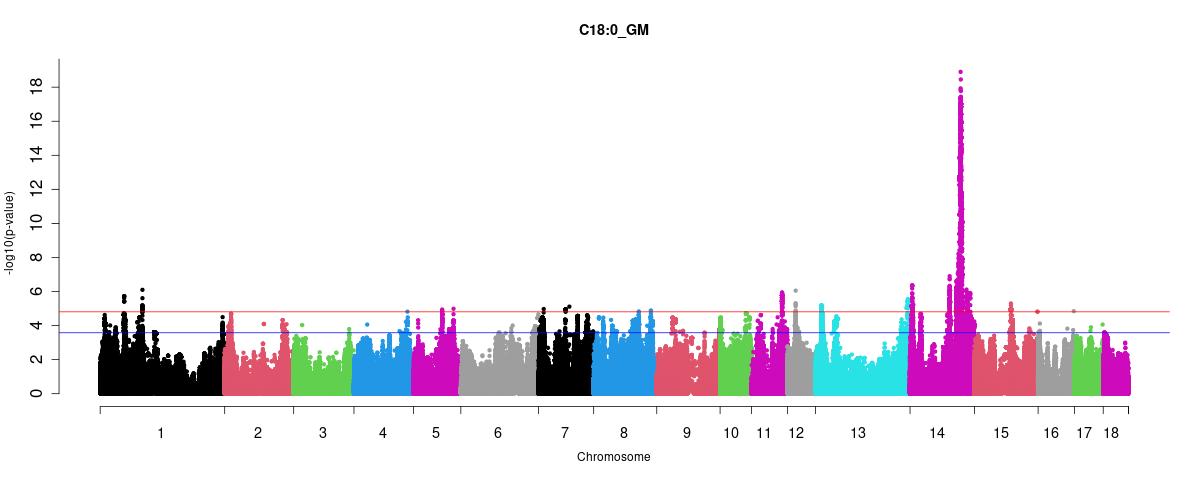

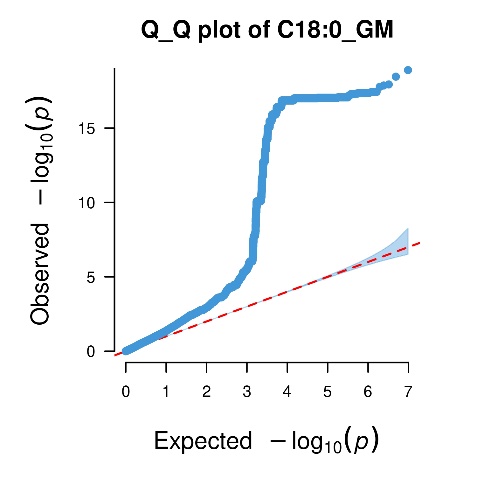


(S)


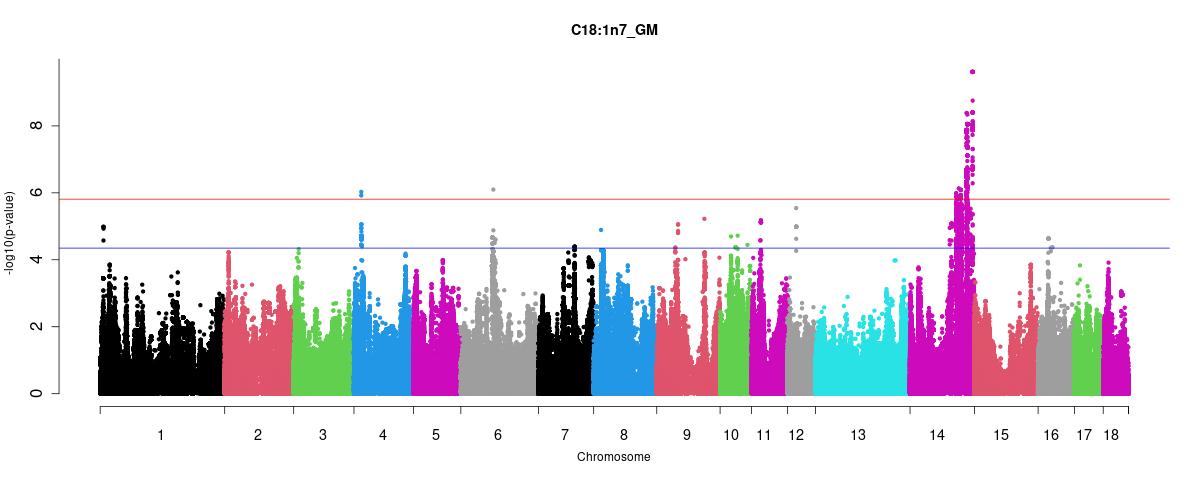

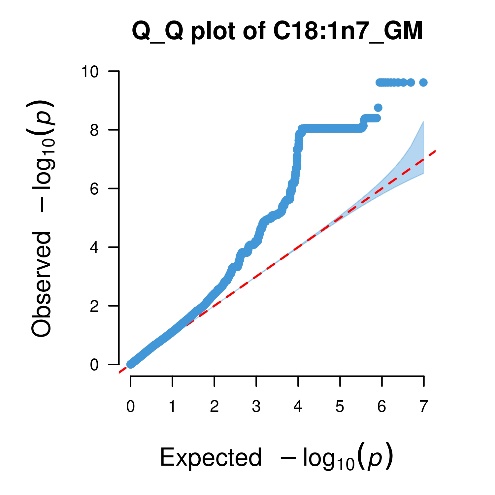

Supplement: Supplementary file 4 — Additional file 4: Figure S1. Manhattan plots and quantile–quantile plots representing the p-values profiles corresponding to the association analysis between fatty acid traits and SNPs,for plasma C16:0for plasma C18:0/C16:0for plasma N6for liver C20:4n-6/C20:3n-6for liver C20:1n-9/C18:1n-9for adipose C20:4n-6/C20:3n-6for adipose C16:0/C14:0for adipose C20:0/C18:0for adipose C18:1n-7for adipose C18:1n-9for adipose C14:0for adipose C22:4n-6for adipose C18:1n-9/C18:0for adipose C16:1n-7/C16:0for muscle C20:4n-6/C20:3n-6for muscle C16:0/C14:0for muscle SFAfor muscle C18:0 andfor muscle C18:1n-7. Red line indicates those SNPs that are below the genome-wide significance threshold. [file 12711_2024_933_MOESM4_ESM.docx]
